# Retrosplenial cortex maturation aligns with the transition from infant forgetting to persistent memory

**DOI:** 10.1016/j.celrep.2026.117768

**Published:** 2026-08-20

**Authors:** Benita Jin, Abigail L. Yu, Michael W. Gongwer, Brian P. Kearney, Alfonso Darmawan, Lilit Ohanian, Lucinda Holden-Wingate, Bryan Le, Yuka Nakayama, Sophia A. Rueda Mora, Anish Velaparthi, Laura A. DeNardo

**Affiliations:** 1 Department of Physiology, University of California, Los Angeles, Los Angeles, CA 90095, USA; 2 Molecular Cellular and Integrative Physiology Interdepartmental Program, University of California, Los Angeles, Los Angeles, CA 90095, USA; 3 Neuroscience Interdepartmental Program, University of California, Los Angeles, Los Angeles, CA 90095, USA; 4 Medical Scientist Training Program, University of California, Los Angeles, Los Angeles, CA 90095, USA; 5 Department of Neurobiology, University of California, Los Angeles, Los Angeles, CA 90095, USA; 6 These authors contributed equally; 7 Lead contact

## Abstract

Memories formed early in life are rapidly forgotten, whereas those formed later persist. Recent work revealed that physical traces of infant memories endure in the brain but are inaccessible to natural recall. However, the developmental processes regulating this transition remain unclear. Here, we performed a brain-wide developmental screen, revealing age-dependent shifts in regional activation patterns that parallel the emergence of stable memory expression. We focused on the retrosplenial cortex (RSP)—a critical region for adult memory consolidation with poorly understood developmental roles—as a candidate whose refinement may influence memory recall. Persistent memory aligned with increased reactivation of RSP ensembles and dendritic spine refinement. Reactivation of RSP infant learning ensembles a month, but not a week, after learning reinstated forgotten memories, coinciding with dendritic spine refinement on those cells. These findings reveal the developmental trajectory of the RSP, uncovering key maturation features aligning with the transition from forgetting to remembering.

## In brief

Infant memories leave enduring brain traces despite natural forgetting. Jin et al. show that developmental refinement of retrosplenial cortex ensembles and dendritic spines parallels persistent memory emergence. Delayed reactivation of infant RSP ensembles reinstates forgotten memories, highlighting RSP maturation as a key feature accompanying developmental transitions in memory accessibility.

## Graphical abstract

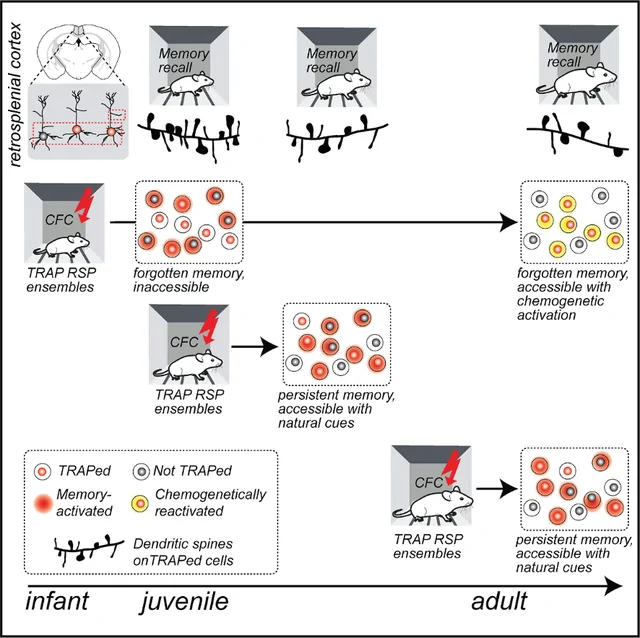

## INTRODUCTION

Infantile amnesia describes a process by which episodic memories formed early in life are rapidly forgotten.^1–3^ In contrast, memories formed later can last for decades. In humans, memories become persistent around age five,^4^ but infantile amnesia is not a uniquely human phenomenon.^5,6^ In rodents, episodic memories become persistent during the fourth postnatal week.^7–11^ Although early-formed memories cannot be consciously recalled, they can influence later experiences by promoting the maturation of learning systems and altering mechanisms of memory encoding.^12–14^ Their continuing influence through development suggests that early-formed memories leave an enduring trace in the brain.

Prior work confirmed the existence of lasting infant memory traces, showing that infant memories are not erased but instead fail to be retrieved. Forgotten memories leave a molecular trace in the amygdala.^15^ Providing strong reminders of the original memory^13,16,17^ or optogenetically reactivating infant memory ensembles in the hippocampus^10^ can recover lost infant memories. Importantly, psychiatric risk factors including early life adversity and maternal immune activation prematurely close the infantile amnesia window,^18,19^ suggesting that infantile amnesia might protect against the emergence of behavioral disorders later in life.^7,20^ Despite their importance for brain and behavioral development, the processes by which infant memories are stored and how they become inaccessible remain poorly understood. If infant memory traces persist in the hippocampus and can be artificially retrieved, what prevents their natural retrieval?

In adults, memory encoding in the hippocampus is followed by a period of consolidation, during which memories become embedded in distributed brain networks for stable, long-term storage.^21^ Memory encoding involves the allocation of highly excitable neurons into neuronal ensembles that are necessary and sufficient for later recall. Both hippocampal and cortical neurons are allocated to a memory ensemble during learning, but cortical memory networks stabilize across a longer time frame.^21,22^ This process requires hippocampal communication with an extended network involving the prefrontal cortex, anterior thalamic nuclei, and the retrosplenial cortex (RSP).^21–23^ Over time, offline hippocampal reactivations and synaptic plasticity strengthen the cortical memory network and its role in memory recall.^22^

Previous studies revealed that infantile amnesia is associated with immature hippocampal synapses and inhibitory circuits^13,14,24^ and naturally elevated hippocampal neurogenesis,^9^ findings that suggested extensive hippocampal circuit remodeling contributes to infant forgetting. Although hippocampal circuit remodeling is likely to disrupt hippocampal-cortical interactions, little is known about the functional maturation of cortical areas involved in memory consolidation and how this impacts memory storage and retrieval across development. While the protracted postnatal refinement of cortical connectivity^25–27^ may gate the stabilization and expression of infant memories, this remains unexplored.

Here, we used TRAP2 mice to perform a brain-wide developmental screen, identifying key regions—including the RSP—whose memory-evoked activation patterns change significantly across the transition from forgetting to remembering. Chronically reactivating RSP memory ensembles for a week after learning failed to enhance memory in infants but had graded effects across subsequent stages of development. These developmental changes in RSP function were accompanied by enhanced RSP ensemble reactivation during memory recall in adults and juveniles, but not in infants. Whereas dendritic spines were dense on infant-TRAPed RSP cells regardless of learning, we observed significant synaptic sparsening with age as well as learning-related increases in spine density between juvenile and adult stages. Reactivation of infant learning-activated RSP ensembles weeks later recovered forgotten memories, a process accompanied by spine sparsening on those cells. Together, these findings suggest that developmental changes in the reactivation of memory-encoding RSP neurons and the synaptic organization of the RSP may contribute to infant forgetting and the later accessibility of latent infant memories. More broadly, we define the timeline of RSP maturation as it relates to memory persistence.

## RESULTS

### A brain-wide screen to identify neural signatures of infantile amnesia

We modeled infantile amnesia in mice using an established protocol.^8–10^ We performed contextual fear conditioning (CFC) when mice were infants (postnatal day (P) 17), juveniles (P25), or adults (P60). We tested the strength of their fear memory either 1 day (d) or 7 d after CFC, in separate cohorts of mice, by placing mice in the original training context and measuring freezing behavior (Figure 1A). While mice of all ages froze robustly during CFC and the 1 d memory retrieval test, mice trained as infants froze significantly less than juveniles and adults during a 7 d memory retrieval test (Figure 1B).

Using this behavioral model, we designed a screen to investigate how brain-wide, memory-related cellular activation changes across development. We labeled activated neurons using Targeted Recombination in Active Populations (TRAP2: *Fos*^*2A-iCreERT2*^),^28,29^ in which the Fos promoter drives the expression of tamoxifen-inducible Cre recombinase. We crossed TRAP2 to the Ai14 Cre reporter line^30^ to label TRAPed neurons with a red fluorophore (tdTomato). We performed CFC in infant, juvenile, or adult *TRAP2;Ai14* mice alongside age-matched non-shocked (NS) control mice, which were placed in the chamber but did not receive shocks. One day later, we TRAPed memory-activated neurons by pairing re-exposure to the training context with a tamoxifen injection. Allowing a week for tdTomato expression, we then used tissue clearing, light-sheet fluorescence microscopy, and machine learning-based pipelines to quantify the number of TRAPed cells per brain region (Figure 1C; Table 1; Table S1). Fear-conditioned mice of all ages froze significantly more than NS controls (Figure 1D). We TRAPed 1 d retrieval rather than CFC to capture memory-related processes without capturing the experience of foot shocks, which strongly activate many brain areas.^28^

To determine how brain-wide memory-related activation changes over development, we first normalized CFC brains to age-matched NS controls by calculating a “learning index,” a metric we developed to represent the deviation between the observed number of TRAPed cells in CFC mice and what was expected based on the age-matched NS controls (see Methods). Using these normalized values, we generated volcano plots to identify brain regions that had significant differences in the number of TRAPed cells in CFC versus NS mice for each age group (Figures 1E–1G). In infants, we observed greater memory-related activation in subcortical areas including the dorsal premammillary nucleus (PMd), anterior hypothalamic nucleus (AHN), postpiriform transition area (TR), and anterior group of the dorsal thalamus (ATN). In contrast, in juveniles and adults, we observed increased activation of association cortices with known roles in memory consolidation and remote memory retrieval, including the RSP, temporal association areas (TEa), anterior cingulate area (ACA), ectorhinal cortex (ECT), and basolateral amygdala (BLA).

As many brain regions are anatomically and functionally heterogeneous, we next evaluated within-region variations in age-specific memory activation. To achieve this, we segmented brains into 10 μm voxels, with each voxel representing the mean cell density of the surrounding ~500 μm. We first performed voxel-based comparisons between CFC and NS animals within each age group (Figure S1). Compared to NS controls, CFC infants had higher activation in the hypothalamus. In contrast, CFC juveniles and adults had higher activation in subregions of the cortex, including RSP, amygdala, and hippocampal formation. In all ages, NS mice showed higher activation in areas associated with sensation and movement (SSp, MOp, caudate), consistent with their lower levels of freezing (Figure S1; Figure 1D).

To determine how regional activation patterns change across development, we normalized each voxel to that of age-matched NS controls by calculating the learning index as before and then performed voxel-wise statistical comparisons across ages (Figure 1H). We first focused on voxels that had a significantly higher learning index in juvenile and adult brains compared to infant brains (dark purple voxels, Figure 1H), as this aligns with the onset of memory persistence. We observed this effect in the piriform cortex (PIR), midline thalamic nuclei (MTN), ATN, ECT, TEa, subregions of the subiculum (SUB, PRE, POST), and RSP. In contrast, we found that voxels in the hypothalamic zona incerta (ZI) and motor areas of the midbrain (MBmot) had a higher learning index in infant mice compared to older animals (gold voxels, Figure 1H). Juvenile mice had a uniquely high learning index in several posterior cortical regions and the lateral septum (LSX; pink voxels, Figure 1H). Adult mice had uniquely high learning indices in prelimbic and infralimbic cortices (PL, ILA) as well as the hypothalamic periventricular zone (PVZ), medial preoptic nucleus (MPN), and mammillary body (MBO; teal voxels, Figure 1H). Together, these results indicate that, during the maturation of memory systems, individual regions and subregions follow distinct developmental timelines and highlight key areas that may differentially regulate memory storage, consolidation, and retrieval across development.

### Network-level analysis of brain-wide TRAPed cells and functional manipulations of RSP memory ensembles point to RSP as a key candidate area for further investigation

To gain further insight into how coordinated activity across regions may contribute to memory recall at each age, we conducted a partial least-squares (PLS) analysis^31^ of brain-wide TRAPed cells. We identified latent variables that differentiated CFC mice from NS controls during retrieval. Bootstrap ratios (|BSR| > 2.0) of salience values revealed distinct sets of reliable positive contributors with relatively more TRAPed cells in CFC mice. Regions with negative bootstrap ratios exceeding the threshold reflected relatively more TRAPed cells in NS controls (Figures 2A–2C). In infant mice, we observed a modest, relatively centered distribution of BSR values, suggesting weak differentiation between CFC and NS mice at this age. Only a few regions exhibited large magnitude BSRs, including the perirhinal cortex and the ATN, and to a lesser extent, the hippocampal subregions CA1 and dentate gyrus (DG) (Figure 2A). In juveniles and adults, we observed overall larger magnitude BSR values, including in several cortical areas, including the RSP, frontal pole (FRP), ECT, and TEa, as well as the lateral amygdala (Figures 2B and 2C), some of which play key roles in memory consolidation and retrieval. These regions were not present in infants (Figure 2A), suggesting that, beginning in juvenile stages, memory retrieval reliably engages a network of neocortical, retrohippocampal, and amygdalar areas. These results suggest that the developmental transition from forgetting to remembering is accompanied by the emergence of a stable, distributed retrieval network spanning key cortical areas.

Overall, our developmental screen provided convergent evidence that brain regions known to play key roles in memory consolidation and long-term storage in adults-including RSP, TEa, ECT, LA, and BLA-do not exhibit memory-selective activation in infant mice. Instead, memory traces in infants are characterized by a unique network of olfactory, hypothalamic, and hippocampal regions. Although the RSP has well-established roles in memory consolidation and long-term memory retrieval in adults,^32–37^ its role in memory in the developing brain remains poorly understood. Moreover, the strong interactions between the RSP and the hippocampus during memory consolidation^38–40^ suggest that RSP maturation may influence whether memories remain accessible or fail to stabilize and become inaccessible to natural reminder cues.

To determine if the RSP was a compelling candidate for further developmental investigations, we used chemogenetics to examine whether the functions of RSP memory ensembles change across development. In adults, activating RSP memory ensembles is sufficient to drive memory recall, and inhibiting RSP impairs remote memory recall, and offline activity within RSP networks is important for memory consolidation.^21,34,41–46^

Because we observed 1 d retrieval-related increases in RSP TRAPed cells selectively in juveniles and adults, we first examined the effects of inhibiting RSP at this timepoint. Although expression time prevented us from selectively inhibiting TRAPed ensembles during the 1 d test, we broadly inhibited RSP in each age group by microinjecting an AAV expressing the inhibitory DREADDs (designer receptor exclusively activated by designer drugs) hM4Di^47^ and delivering the ligand clozapine-N-oxide (CNO) 30 min prior to the 1 d retrieval test. This manipulation had no effect on freezing behavior in any age group (Figures S2A–S2C). These findings are consistent with prior studies showing that RSP has elevated Fos expression at this timepoint, but is not required for recent memory in adults.^48^ This is also consistent with the notion that in the cortex, early neuronal tagging is followed by a slower maturation of the cortical memory trace, a process that integrates the RSP into a distributed memory network.

We next tested whether activating TRAPed RSP neurons during the 7 d test could enhance memory. Because developmental changes in RSP memory functions had not been previously investigated, we began with a comprehensive manipulation—activating 1 d-TRAPed ensembles both offline following 1 d retrieval and acutely immediately before 7 d recall (Figure 2D). This allowed us to assess whether activation could enhance 7 d memory retrieval in each age group. We injected into RSP AAVs expressing the Cre-dependent excitatory DREADD hM3Dq^47^ or a fluorophore control. We TRAPed RSP neurons activated by 1 d memory retrieval, thereby driving the expression of hM3Dq in those cells. We performed offline reactivation of the TRAPed ensembles by administering CNO daily and then administered a final dose of CNO 30-min prior to the retrieval test on day 7 (Figure 2D). Histology confirmed that hM3Dq-mCherry was expressed in a similar location within RSP and to a similar extent across age groups (Figure S2D).

Activating TRAPed RSP neurons affected fear memory retrieval in a graded developmental fashion. Freezing during CFC and 1 d retrieval indicated fear learning occurred in both control and hM3Dq animals at all ages (Figure 2E). In infant mice, both control and hM3Dq groups froze minimally during 7 d retrieval, indicating that enhancing activity in RSP memory ensembles in infant mice cannot prevent infantile amnesia (Figures 2E and 2F). We observed a small memory enhancement in juveniles—chemogenetic activation prevented the extinction-like decrease in freezing we observed in controls between 1 d and 7 d tests (Figures 2E and 2F). Adult hM3Dq-expressing mice froze significantly more than controls during 7d retrieval (Figures 2E and 2F). Together, these data suggest that the memory-promoting functions of the RSP emerge between infant and juvenile stages but continue to evolve between juvenile and adult stages.

To investigate potential circuit-level differences underlying the distinct, age-specific effects of reactivating RSP ensembles, we sacrificed the mice after the 7 d retrieval test and quantified the number of Fos+ neurons in brain regions with direct anatomical connections or strong functional connectivity with RSP.^26,33,49–54^ Compared to adults, infants had significantly fewer Fos+ neurons in the dorsal caudate putamen (CPd), anterior thalamic nuclei (AM, AV, LD and LP), and the anterior pretectal nucleus (APN), a midbrain motor nucleus (Figure S3). All these areas receive projections from the RSP,^55–57^ suggesting broad circuit engagement changes over development. Based on these results and the convergent findings in our screen (Figure 1), we chose to further examine the role of RSP in memory retrieval and consolidation across development.

### Reactivation of TRAPed RSP neurons during memory retrieval increases with age

Having established that chemogenetic reactivation of RSP ensembles can enhance memory recall in an age-dependent manner, we next asked whether endogenous reactivation of these ensembles during retrieval differs across development. Memory persistence has been ascribed to the reinstatement of patterns of neuronal ensemble activity that were established during initial learning.^58,59^ In the adult RSP and hippocampus, immediately early gene mapping studies have shown that neuronal ensembles activated during learning are reactivated during subsequent recall,^28,60^ and that the extent of learning ensemble reactivation predicts retrieval success.^5^ Because infantile amnesia is characterized by impaired memory retrieval, these findings raise the possibility that memory traces are encoded in RSP but are not efficiently re-engaged at later time points.

To test this, we TRAPed RSP neurons activated during CFC and then stained for Fos following the 7 d retrieval test (Figures 3A–3C). Fear-conditioned juvenile and adult mice had significantly higher proportions of reactivated RSP neurons compared to both infants and age-matched NS controls (Figure 3D). We observed a significant effect of age on the number of TRAPed cells—both CFC and NS infants had more than juveniles and adults, respectively (Figure 3E). Surprisingly, all age groups had more Fos+ cells in CFC mice compared to NS controls (Figure 3F). Together, these findings suggest that re-exposure to the conditioned context in infants is associated with broad RSP activation (Figure 3F), but reduced reactivation of learning-activated ensembles (Figure 3D).^24,61^

Given these results, we investigated whether CFC-TRAPed RSP neurons are necessary for 7 d retrieval in adulthood. To test their acute function during memory recall, we chemogenetically inhibited CFC-TRAPed RSP neurons during 7 d retrieval in adult mice (Figures S4A–S4C). Since pharmacological blockade of synaptic activity in the RSP can prevent memory consolidation in adults,^62,63^ we separately examined whether the synaptic activity of CFC-TRAPed cells is necessary for 7 d retrieval. To do so, we expressed tetanus toxin—which potently blocks synaptic release—in CFC-TRAPed RSP neurons^64^ (Figures S4D–S4F). We observed no significant difference in freezing levels during the 7 d retrieval test in either experiment, indicating that the activity of learning-activated RSP cells is not necessary for 7 d memory recall. Together, these findings indicate that in the adult RSP, CFC-activated cells may be part of a distributed memory network that is capable of driving recall when artificially activated but whose activity is not required for natural retrieval a week after learning. Alternatively, slower plasticity mechanisms may drive an emergent role for the RSP in remote memory retrieval.^65,66^

Prior work showed that reactivating infant memory encoding ensembles in the DG could recover lost infant memories.^10,67^ Given these findings, and the established role of hippocampal-RSP interactions in memory consolidation,^38–40^ we also investigated reactivation of learning-activated DG neurons by comparing CFC and NS mice across development (Figure S4G). CFC mice exhibited greater DG ensemble reactivation than NS controls at all ages (Figure S4H). Notably, infants had significantly more TRAPed DG neurons than juveniles or adults in both CFC and NS groups (Figure S4I), consistent with previous reports of broader learning-related neuronal recruitment in the developing hippocampus.^24^ In contrast, only juveniles and adults displayed memory-associated increases in Fos expression during the retrieval test, whereas infant CFC and NS mice exhibited comparably low Fos levels (Figure S4J).

These findings suggest that learning-related ensemble allocation and retrieval-associated Fos induction in the DG are dissociable developmental processes. Thus, although infant DG neurons can be recruited during learning and later reactivated in a learning-specific manner, retrieval-associated Fos induction in the broader DG network is reduced after infantile amnesia has set in. These findings further suggest that there are developmental differences between hippocampal and cortical memory-related circuit activation.

### Developmental and learning-related changes in dendritic spines on TRAPed RSP neurons

Neuronal reactivation depends in part on synaptic connectivity, leading us to ask whether developmental changes in RSP ensemble reactivation are accompanied by maturation of synaptic inputs. Dendritic spines are the principal sites of excitatory synaptic input in the brain and undergo extensive remodeling during postnatal development. However, the developmental trajectory of spine maturation in RSP, and how it aligns with the emergence of persistent memory, remains unknown.

We investigated RSP spine density and morphology in infants, juveniles, and adults. We crossed TRAP2 to MORF3 mice that stochastically express a V5 tag in a subset of Cre+ neurons.^68^ This bright, sparse labeling enables detailed quantification of dendritic spines. We TRAPed RSP neurons during CFC and analyzed spine density 7 d later (Figures 4A–4C).

We observed significant effects of age, CFC, and their interaction on RSP spine density (Figure 4D). In NS control mice, we observed a progressive decrease in RSP dendritic spines across groups (Figure 4D). Since we measured spines 7 d after TRAPing, when mice were P24, P32, and P67, respectively, this reveals a substantial reduction in spine density between juvenile and adult stages. In CFC mice, infant TRAPed cells had slightly lower spine density compared to NS controls, juveniles had similar spine density to NS controls, but adults had significantly higher spine density than NS controls (Figure 4D). These findings reveal the time course of RSP spine development and suggest that differences in spine density do not determine reactivation rates per se. These findings also suggest that in adults, CFC may preferentially recruit spinier RSP neurons or promote structural plasticity compared to context exposure alone.

Although we did not observe learning-related differences in overall spine density in juveniles, we investigated whether morphologically defined spine subtypes may exhibit learning-dependent structural plasticity in juveniles or other age groups. We used established metrics^69,70^ to classify spines as stubby, thin, or mushroom-shaped (Figures 4E–4G). Stubby spines may be immature or may represent a transitional state after experience in adults; thin spines are thought to be highly plastic, and mushroom spines are thought to contain synapses that have undergone experience-dependent strengthening.^71,72^ Seven days after CFC, juveniles had more thin spines (Figure 4F), whereas adults had more mushroom spines (Figure 4G), consistent with ongoing synaptic remodeling between juvenile and adult stages.

### Reactivation of infant-TRAPed RSP ensembles weeks after learning can recover lost memories

The developmental increases in RSP ensemble reactivation (Figure 3) and the timeline of RSP dendritic spine maturation (Figure 4) raised the possibility that infant memory ensembles persist in the RSP but are not efficiently recruited for retrieval early in development. To test this, we examined whether chemogenetic activation of infant CFC-TRAPed RSP ensembles could promote memory retrieval after a delay, when cellular and synaptic maturation had reached adult-like levels. We injected an AAV encoding a Cre-dependent hM3Dq or a fluorophore control into RSP of TRAP2 mice and then TRAPed RSP neurons during CFC at P17. Either 7 or 33 d later, in separate groups of mice, we delivered CNO 30 min before a memory retrieval test (Figure 5A). To examine the interaction between our manipulation and presentation of memory cues, we tested mice in both the conditioned context (context A) and in an altered context (context B) that was in a distinct room and had altered floor, wall decorations, and scent (Figure 5A). As before, histology confirmed that hM3Dq-mCherry was targeted to RSP (Figure S5A).

We observed no effect of hM3D-mediated activation at the 7 d time point, although we did observe that mice in both groups froze even less in context B compared to A (Figure 5B), suggesting that the remnants of the infant memory are context-sensitive. After a 33-day delay, however, we observed a strong increase in freezing behavior in hM3D-expressing mice, an effect that was stronger in context A versus context B (Figure 5C). Reactivating infant-TRAPed neurons in ACA or LSX did not enhance freezing 33 d later (Figure S5B), supporting a specific role for the RSP. Together, these results indicate that reactivating infant learning-related RSP ensembles can recover latent infant memories after extended developmental maturation, but only when the original cues are present.

We next asked whether long-term accessibility of infant memories was associated with developmental changes in RSP dendritic spine organization. To test this, we quantified dendritic spine density on P17 CFC-TRAPed or NS-TRAPed RSP neurons after a 33-day delay (Figures 5D and 5E) and compared these measurements to spine density after a 7 d delay (Figures 4 and 5F–5I). Both infant CFC- and NS-TRAPed RSP neurons had significantly lower spine density 33 days later compared to 7 days later (Figure 5F), indicating substantial developmental synaptic sparsening over this interval. Notably, this structural refinement coincided with the emergence of successful infant memory retrieval following RSP ensemble reactivation. We also found that CFC mice showed a developmental increase in stubby spines between the 7 and 33 d delays (Figures 5G–5I). Together, these findings reveal developmental remodeling of RSP dendritic spine organization that parallels the later accessibility of infant memories.

## DISCUSSION

Even though very early experiences cannot be explicitly recalled, they can profoundly impact the rest of our lives.^5,7,14,73^ Recent work sheds light on this paradox, showing that infant memories are not erased but rather inaccessible by natural cues.^10,13,15–17^ However, the mechanisms underlying this inaccessibility are poorly understood, especially in cortical areas, which have a prolonged developmental timeline. To investigate this, we conducted a brain-wide screen to identify developmental changes in memory circuits. We observed widespread changes in regional activation patterns that coincided with the onset of persistent memory. In juveniles and adults, but not in infants, we observed memory-driven increases in RSP activation. Likewise, RSP neurons activated during CFC were more likely to be reactivated during recall in juveniles and adults. We also found that chronically reactivating TRAPed RSP ensembles during the week following CFC enhanced memory in adults and somewhat in juveniles, but not in infants. In contrast, acute activation of infant-TRAPed RSP ensembles more than a month after CFC successfully reinstated infant fear memories, and this was accompanied by dendritic spine sparsening in learning-activated cells of the RSP. Together, these findings define the developmental trajectory of the RSP during the emergence of persistent memory and reveal that learning-activated RSP ensembles encode forgotten infant memories.

In adults, the RSP plays a key role in memory consolidation and long-term memory retrieval.^65,66^ Long-range interactions between RSP, ATN, and the prefrontal cortex are essential for memory consolidation.^74^ Within the RSP, neuronal and synaptic activity during learning are required for memory consolidation.^62,75^ Learning drives Fos expression in the RSP, and memory retrieval drives the reactivation of the learning-activated neurons.^34,37^ RSP dendritic spine turnover and spine clustering are important for stabilizing lasting memories.^76^ Artificial activation of learning-activated RSP neurons drives memory recall and, when done during sleep, can accelerate memory consolidation.^34,37^ By tracking developmental changes in the cellular and synaptic properties of learning-activated RSP neurons, and reactivating these cells in different developmental windows, our work provides insight into how RSP memory functions mature across transitions in memory persistence.

Based on our findings, one possible reason why infant memories fail to be retrieved is that contextual cues fail to sufficiently or specifically reactivate learning-activated ensembles during retrieval. During early postnatal development, cortical neurons exhibit higher intrinsic excitability, receive weaker inhibition, and have enhanced NMDAR-mediated Ca^2+^ influx due to a higher proportion of GluN2B-containing receptors.^77,78^ Together, these properties likely reduce the threshold for neuronal activation and Fos expression during infant learning, leading to broad neuronal allocation. As cortical neurons become less excitable and inhibition strengthens across the transition from infant to juvenile stages, the same contextual cues may be less effective at selectively re-engaging these broadly allocated neurons during retrieval, which could contribute to retrieval failure. Consistent with this, during CFC we observed more TRAPed cells in the infant RSP compared to juveniles and adults, yet higher memory-related reactivation rates in juveniles and adults (Figure 3), results that align with prior studies of the hippocampus.^24,67^

Our whole-brain analyses of TRAPed cells during 1 d memory retrieval provide insight into how broader memory networks mature. Juveniles and adults had more TRAPed RSP cells in CFC mice compared to NS controls. In contrast, infants had similar numbers of TRAPed RSP cells in CFC and NS mice (Figures 1 and 2). Notably, other memory-relevant regions matured along distinct timelines, including regions that are directly connected with RSP.^21,38,42,43,54,66–73^ Juveniles and adults exhibited learning-dependent activation of regions that are critical for memory consolidation and retrieval, including TEa, ECT, and LA. In contrast, CFC infants had more learning-dependent activation in hypothalamic nuclei including PMd and AHN (Figures 1 and 2), regions that are associated with freezing behavior, but have not been studied in development.^79,80^ The prefrontal cortex and midbrain, regions that may be important for increases in memory complexity or specificity that emerge later in development,^5,24,81^ continued to change their activation between juvenility and adulthood. These results build a foundation for defining how changes in the functional organization of brain-wide memory networks influence memory persistence.

Our findings also reveal how interactions between developmental RSP synaptic refinement programs and learning align with the transition from infantile amnesia to persistent memory. While we observed that adult CFC-TRAPed cells had more spines compared to NS-TRAPed cells, previous work did not observe learning-dependent increases in spine number in the RSP,^76,82^ with the caveat that they did not focus specifically on learning-activated cells. Our observation may reflect CFC-driven structural plasticity or activation of spinier RSP neurons.

In juveniles, we observed reduced spine density between CFC and 7d recall alongside a learning-related increase in thin spines and successful reactivation of learning-activated RSP neurons (Figures 3 and 4). Thin spines have relatively weak NMDAR-containing excitatory synapses. They can form rapidly in response to synaptic activity and undergo experience-dependent strengthening through addition of AMPARs.^71^ With these properties, thin spines have been proposed to have a greater capacity for plasticity compared to mushroom spines.^71^ Together, our results suggest that in juveniles, the cellular and synaptic properties of the RSP may already support adult-like patterns of neuronal allocation and reactivation (Figure 3). However, ongoing synaptic refinement and plasticity between juvenility and adulthood may still influence the rate of consolidation or memory stability. This aligns with our finding that reactivating juvenile ensembles promotes memory recall less efficiently compared to adults (Figure 2), and further suggests that neuronal allocation to memory ensembles (Figure 3) and synaptic remodeling associated with learning (Figure 4) are separable processes that mature along distinct developmental timelines.

In the infant neocortex, synapses are more labile, Fos expression is more widespread, and the molecular programs that underlie learning-driven synaptic plasticity in adults are instead engaged in developmental refinement.^83^ Consistent with this, we observed higher spine density in both CFC and NS infant mice (Figure 4). Over the course of a month, however, these same neurons exhibited extensive spine loss (Figure 5). Notably, reactivating these neurons weeks after learning significantly increased freezing behavior, but only in the conditioning context. This suggests that contextual information can interact with the chemogenetic activation of these neurons to influence retrieval behavior. Moreover, these results reveal how synaptic sparsening between infancy and adulthood aligns with the emergence of memory-promoting effects of RSP ensembles. Importantly, our static readout of spine density cannot distinguish between states of synaptic instability versus states of active developmental pruning of stable synapses. Moreover, we are blind to the identity of remodeled connections. Future research can examine which RSP connections are pruned and how developmental changes in spine dynamics align with changes in memory persistence and accessibility.

Hippocampal inputs to the RSP are critical for memory consolidation, and RSP inputs to the hippocampus are critical for remote memory retrieval in adults. Prior work showed that optogenetic reactivation of learning-activated hippocampal ensembles as early as 15 days after CFC (~P32) can reinstate forgotten infant memories.^10^ These findings align with our observation that infant memories become accessible following the reactivation of learning-activated RSP sometime between P24 and P50 (Figure 5). In developing rodents, bidirectional RSP-hippocampal connections reach adult-like densities by P12,^26,27^ and in humans, resting state RSP-hippocampal functional connectivity matures before adolescence,^84^ but how these changes impact memory persistence remains unknown. Thus, an important future direction will be to determine how the maturation of hippocampal-RSP connections influences memory persistence, as well as the memory-promoting effects of RSP ensemble reactivation across development.

### Limitations of the study

Scientists are just beginning to understand the role of the developing brain in learning and memory. For this reason, we prioritized using a brain-wide screen and chemogenetic manipulations to identify understudied regions of interest whose developmental programs align with the emergence and maturation of persistent memories. Our approach targeted Fos+ cells following a contextual fear memory recall. Although Fos is widely used for labeling and genetically accessing activated neuronal populations, the precise relationships between neuronal activity and Fos expression are not well defined for every brain region. Furthermore, Fos provides a snapshot of activated populations, and chemogenetic activators broadly alter neuronal excitability across a few hours. Consequently, these approaches cannot resolve the temporal dynamics of neuronal activity that support memory encoding and recall, nor how they change across development. Similarly, while our static descriptions of dendritic spine morphology provide key insights into the timing of RSP synapse development, they cannot reveal how development shapes dynamic synaptic reorganization during learning and memory consolidation. Future studies should capture the longitudinal dynamics of RSP activity and synaptic connectivity and use these to guide causal, temporally precise manipulations. These approaches can provide a more nuanced understanding of the neural processes that drive transitions between infant forgetting and persistent memory.

## RESOURCE AVAILABILITY

### Lead contact

Requests for further information and resources should be directed to and will be fulfilled by the lead contact, Laura A. DeNardo (ldenardo@ucla.edu).

### Materials availability

This study did not generate new, unique reagents.

### Data and code availability

- All data reported in this paper will be shared by the lead contact upon request.
- All original code has been deposited at Zenodo and is publicly available as of the date of publication. DOIs are listed in the key resources table.
- Any additional information required to reanalyze the data reported in this paper is available from the lead contact upon request.
- Custom data analysis scripts were coded in MATLAB or Python and are available on Zenodo (https://doi.org/10.5281/zenodo.21283955; https://doi.org/10.5281/zenodo.21283773).

## STAR★METHODS

### EXPERIMENTAL MODEL AND STUDY PARTICIPANT DETAILS

#### Animals

Female and male C57BL6/J mice (JAX Stock No. 000664) or TRAP2;Ai14 mice (JAX Stock Nos. 030323 and 007914) were group housed (2–5 per cage) and kept on a 12 h light cycle (lights on 7am-7pm). All animal procedures followed animal care guidelines approved by the University of California, Los Angeles Chancellor’s Animal Research Committee.

### METHOD DETAILS

#### Surgery

Animals underwent surgery at P3, P9 or P46. Depending on the experiment, mice were anesthetized through hypothermia (P3 mice) or induced in 5% isoflurane in oxygen until loss of righting reflex (P9 or older) and transferred to a stereotaxic apparatus with rubber ear bars. P9+ mice were maintained under 2% isoflurane in oxygen, and warmed with a circulating water heating pad throughout surgery and eye gel was applied to the animal’s eyes. The mouse’s head was shaved and prepped with three scrubs of alternating betadine and then 70% ethanol. Following a small skin incision, a dental drill was used to drill through the skulls above the injection targets. A syringe pump (Kopf, 693A) with a Hamilton syringe was used for injections delivered at a rate 100nL/min. The syringe was left in the brain for 1 or 7 min (min) following injection for P3 and P9+ mice respectively. For DREADD studies, a small volume of AAV8-hSyn-DIO-hM3D(Gq)-mCherry or AAV-hSyn-hM4D(Gi)-mCherry (Addgene 44361 and 50471 final titer 2 × 10^12^ pp per mL) was injected bilaterally into RSP (AP: −2.7, ML: ±0.3, DV: 1.0). At P3 and P9, this coordinate was scaled using Neurostar StereoDrive software (Neurostar) which corrects for head size, orientation, and tilt. To compensate for age-dependent changes in cortical volume, we made proportional adjustments to the volume of injected virus based on previous developmental studies^83^: 0.15 μL for P3, 0.2 μL for P9, and 0.4 μL for P46 mice. Control mice received injections of age-matched volumes of AAV1-pCAG-FLEX-eGFP-WPRE (Addgene 51502, final titer 1 × 10^13^ pp per mL) into RSP. For tetanus toxin experiments in adults, we injected AAV-DJ-CMV-DIO-EGFP-2A-TENT (Stanford Vector Core, 1 × 10^13^ pp per mL) using the same methods. For pain management mice received 5 mg/kg carprofen diluted in 0.9% saline subcutaneously. Mice received one carprofen injection 30 min prior to surgery and daily injections for two days following surgery. Following confirmatory histology, samples with mistargeted injection sites were excluded from analysis.

#### 4-Hydroxytamoxifen preparation

4-hydroxytamoxifen (4-OHT; Sigma, Catalog #H6278) was dissolved at 20 mg/mL in ethanol by shaking at 37°C for 15 min and was then aliquoted and stored at −20°C for up to several weeks. Before use, 4-OHT was redissolved in ethanol by shaking at 37°C for 15 min, a 1:4 mixture of castor oil:sunflower seed oil (Sigma, Catalog # 259853 and S5007) was added to give a final concentration of 10 mg/mL 4-OHT, and the ethanol was evaporated by vacuum under centrifugation. The final 10 mg/mL 4-OHT solutions were always used on the day they were prepared. All injections were delivered intraperitoneally (50mg/kg, i.p.).

#### Behavioral assays

Mice were placed in an operant chamber with a shock floor. For context fear conditioning, after exploring the arena for two minutes, mice received 5 1-s, 0.5 mA foot shocks separated by 1 min. On retrieval days, mice were placed in the conditioning chamber for 5 min. For TRAP experiments, mice were wrapped in a soft towel for immobilization and injected with 4-OHT solution immediately following the retrieval session. For quantifying MORF+ cells, learning sessions were for two days, with 3 shocks on day 1 and 2 shocks on day 2. Mice were excluded from the study if freezing during CFC was below 15%, indicating poor learning. Point-tracking of behavioral videos was performed in DeepLabCut.^85^ Locomotion and freezing behavior were analyzed using BehaviorDEPOT.^86^

#### Brain clearing

Two weeks after TRAPing, mouse brains were collected and processed based on the published Adipo-Clear protocol^87^ with slight modifications. Mice were perfused intracardially with 20mL of PBS (Gibco) followed by 4% paraformaldehyde (PFA, Electron Microscopy Sciences) on ice. Brains were hemisected approximately 1 mm past midline and postfixed overnight in 4% PFA at 4°C. The following day, samples were dehydrated with a gradient of methanol (MeOH, Fisher Scientific):B1n buffer (1:1,000 Triton X-100, 2% w/v glycine, 1:10,000 NaOH 10N, 0.02% sodium azide) for 1 h for each step (20%, 40%, 60%, 80%) on a nutator (VWR). Samples were then washed with 100% MeOH 2× for 1 h each and then incubated in a 2:1 dicholoromethane (DCM):MeOH solution overnight. The following day, two washes of 1 h in 100% DCM were performed followed by three washes of 100% MeOH for 30 min, 45 min then 1 h. Samples were bleached for 4 h in 5:1 H2O2/MeOH buffer. A cascade of MeOH/B1n washes (80%, 60%, 40%, 20%) for 30 min each rehydrated the samples followed by a 1-h wash in B1n buffer. 5%DMSO/0.3M Glycine/PTxWH permeabilized tissue for one hour and then again for 2 h with fresh solution. Samples were washed with PTxwH for 30 min and then incubated in fresh PTxwH overnight. The following day two more PTxwH washes lasted 1 h then 2 h. For TRAP2 experiments, samples were incubated in primary RFP antibody (Rockland 600–401-379) at 1:300 in PTxwH shaking at 37°C for 11 days, washed in PTxwH 2 × 1 hour and then 2 × 2 hours, then for two days with at least one PTxwH change per day while shaken at 37°C. Samples were then incubated in secondary antibody Alexa Fluor 647, catalog #A-31573, 1:300, Thermo Fisher Scientific) for 8 days shaken at 37°C. Samples were washed in PTxwH 2 × 1 hour, then 2 × 2 hour, then 2 days with at least one PTxwH change per day while shaken at 37°C. Samples were then washed in 1× PBS twice 1 × 1 hour, 2 × 2 hours and then overnight. To dehydrate samples, a gradient of washes in MeOH:H2O (20%, 40%, 60% and 80%) were conducted for 30 min each, followed by 3 × 100% MeOH for 30 min, 1 h, then 1.5 h. Samples were incubated overnight in 2:1 DCM:MeOH on a nutator. The next day, samples were washed in 100% DCM 2 × 1 hour each. Samples were then cleared in 100% dibenzyl ether (DBE). DBE was changed after 4 h. Samples were stored in DBE in a dark place at room temperature. Imaging took place at least 24 h after clearing. Samples with poor antibody penetration following tissue clearing were excluded from analysis. To limit variation due to behavioral performance in the CFC condition, only mice with at least 35% freezing at 1-day retrieval were included in further analyses.

#### Whole brain imaging

Brain samples were imaged on a light-sheet microscope (Ultramicroscope II, LaVision Biotec) equipped with a sCMOS camera (Andor *Neo*) and a 2×/0.5 NA objective lens (MVPLAPO 2×) equipped with a 6 mm working distance dipping cap. Image stacks were acquired at 0.8× optical zoom using Imspector Microscope v285 controller software. For TRAP2;Ai14 brains, we imaged using 488-nm (laser power 20%) and 640-nm (laser power 50%) lasers. The samples were scanned with a step-size of 3 μm using the continuous light-sheet scanning method with the included contrast adaptive algorithm for the 640-nm channel (20 acquisitions per plane), and without horizontal scanning for the 488-nm channel.

#### DeepCOUNT analysis

##### Image registration & cell segmentation

Whole-brain TRAP counts were obtained using DeepCOUNT as previously described.^88^ Briefly, whole-brain image stacks from the 640nm channel from each brain were segmented in TrailMap^89^ using the TRAP2-Ai14 trained model. Images were registered to the template brain using elastix and transformix using the 488nm autofluorescence channel. MATLAB was used to identify 3D maxima of the transformed probability map. Connected component analysis was used to reduce any maxima that consisted of multiple pixels into a single pixel per cell.

##### Cell quantification

Regional TRAPed cell density was quantified in MATLAB (Mathworks) by counting the number of labeled pixels (i.e., cells) in each brain region, then dividing this pixel count by the total number of pixels in a region. We observed variability in the efficiency of tamoxifen-induced recombination between brains as measured by the total number of TRAPed cells across regions. To account for region- and age-specific differences in recombination efficiency, a linear regression was fitted between the total number of cells in a brain (a proxy for recombination efficiency) and cell counts in a particular region for non-shocked control brains within an age. Using this regression, for each brain region in each CFC mouse, we calculated a predicted TRAP count of a non-shocked age-matched brain based on the total number of TRAPed cells. From this, we calculated the difference between the observed and predicted value for each region. We then divided this number by the distance from the y axis to account for greater deviations from the predicted value as the distance from the y axis increased. This number was also divided by region volume in voxels to account for variable size of brain regions. This final value, which we term the “learning index”, results in a positive value if a brain region has higher counts than the predicted value of a non-shocked brain and a negative value if the counts are lower than would be predicted.

For voxel-based analysis, the same principle was applied. Cell density per voxel was first calculated as a Gaussian distribution of the cell density in a 250um radius surrounding the voxel. In the same way as described above, a learning index was calculated for each voxel to describe whether the observed cell density is higher or lower than what would be predicted in an age-matched non-shocked control. For statistical comparisons between ages as reported in Figure 1H, a one-way ANOVA was performed for each voxel followed by Tukey’s multiple comparisons test, and colored voxels indicate *p* < 0.05 for a particular comparison or set of comparisons. For statistical comparisons between shocked and non-shocked controls, a *t* test was performed for each voxel, and colored voxels indicate *p* < 0.05.

For region-based analysis, regions were defined by a collapsed version of the LSFM atlas in which maximum granularity was balanced with the need to account for slight differences in registration which would lead to inaccurate quantification of small brain regions. This atlas was cropped on the anterior and posterior ends to match the amount of tissue visible in our data. Fiber tracts, ventricular systems, cerebellum, and olfactory bulb were excluded from analysis.

#### Brain slice histology and immunostaining

Mice were transcardially perfused with phosphate-buffered saline (PBS) followed by 4% paraformaldehyde (PFA) in PBS. Brains were dissected, post-fixed in 4% PFA for 12–24 h and placed in 30% sucrose for 24–48 h. They were then embedded in Optimum Cutting Temperature (OCT, Tissue Tek) and stored at −80°C until sectioning. 60 μm floating sections were collected into PBS. Sections were washed 3 × 10 min in PBS and then blocked in 0.3% PBST containing 10% normal donkey serum (catalog #17-000-121, Jackson Immunoresearch) for 2 h. Sections were then incubated in a carrier solution (3% donkey serum in 0.3% PBST) containing the primary antibodies for two nights at 4°C. Two days later, sections were washed 3 × 5 min in PBS and then stained in a carrier solution (5% donkey serum in 0.3% PBST) with secondary antibody for 2 h at room temperature. The following primary antibodies were used: polyclonal chicken anti-GFP (catalog #1020, 1:2000, Aves), monoclonal rabbit anti-RFP (catalog #600-401-379, 1:2000, Rockland), monoclonal rabbit anti-Fos (catalog #226-008, 1:2000, Synaptic Systems), mCherry monoclonal anti-rat (catalog #M11217, 1:500, Thermo Fisher), and rabbit polyclonal anti-V5 (Bethyl). The following secondaries were used: Cy2 polyclonal donkey anti-chicken (catalog #703-225-155, 1:1000, Jackson Immunoresearch), Cy3 polyclonal donkey anti-rabbit (catalog #711-005-152, 1:1000, Jackson Immunoresearch), Alexa 647 polyclonal donkey anti-rabbit (catalog #711-605-152, 1:1000, Jackson Immunoresearch), and Cy3 polyclonal donkey anti-rat (catalog #712-165-153, 1:1000, Jackson Immunoresearch). Sections were then washed 5 min with PBS, 15 min with PBS+DAPI (catalog #D1306, 1:4000, Thermo Fisher), and then 5 min with PBS. Sections were mounted on glass slides using FluoroMount-G (catalog #00-4958-02, Thermo Fisher) and then imaged at 10× with a Zeiss 700 confocal microscope or at 10× on a Leica DM6 B scanning microscope.

#### DREADD and TeNT studies

To manipulate neuronal activity, we used AAVs expressing cre-dependent excitatory DREADDs (AAV-hSyn-DIO-hM3D(Gq)-mCherry) or a fluorophore control (AAV-hSyn-DIO-mCherry), in TRAP2 mice two weeks prior to fear-conditioning, or non-conditional DREADDs (AAV-hSyn-hM4D(Gi)-mCherry) in wildtype mice. In TRAP2 mice, viral expression was driven exogenously by intraperitoneally delivered 4-hydroxytamoxifen, which induced fos-dependent expression of cre-recombinase in the TRAP2 mice, allowing specific tagging and long-term labeling of memory encoding ensembles for weeks later. To determine the effects of chronic activation of TRAPed RSP ensembles during development (i.e., comparing freezing levels between retrieval days 1 and 7), 4-hydroxytamoxifen was intraperitoneally injected immediately after 1-day retrieval to tag ensembles involved in retrieval and consolidation (Figures 1 and 2) or immediately after contextual fear conditioning (Figures 3, 4, and 5).

30 min prior to their first retrieval session, mice were intraperitoneally injected with CNO dissolved in 0.9% saline (1mg/kg, i.p., Fisher Scientific). After the first retrieval session, mice were returned to their home cage. A subset of mice was also tested in an alternate context 3 h after the first testing session and then returned to their home cage. Following the first retrieval test, mice were injected daily with CNO (2 mg/kg, i.p.) for 5 days and then returned to the home cage. 30 min prior to the last retrieval day, CNO (1 mg/kg, i.p.) was injected prior to testing in a similar manner as the first retrieval day. Mice were perfused 1 h after the last retrieval session and their brains collected and processed for Fos immunostaining.

To silence neuronal output, we used an AAV expressing cre-dependent tetanustoxin virus (AAV-DJ-CMV-DIO-eGFP-2A-TeNT) or a fluorophore control (AAV-hSyn-DIO-mCherry), in the RSP in adult TRAP2 mice two weeks prior to fear-conditioning. I.P. administration of 4-hydroxytamoxifen after fear conditioning occurred as described in the above DREADD experiments to induce expression of the TeNT virus or fluorophore control in the cells TRAPed during CFC. The retrieval session occurred 7 days after learning, where mice were placed back into the original context. Freezing was evaluated as previously described.

#### Fos analysis in 2-D sections

##### Alignment

Following Fos immunostaining and imaging as described above, sections were aligned to the Allen Brain Atlas^90^ using DeepSlice,^91^ a deep-learning based algorithm for automatically calculating the position and slicing angle of each section in relation to the atlas. The DAPI channel was used for alignment. Alignments were imported to the ABBA plugin for FIJI^92,93^ (https://biop.github.io/ijp-imagetoatlas/) which was used to manually check the alignment for each section. ROIs from ABBA were then exported to FIJI, and ROIs for regions to be analyzed were manually adjusted to correct for minor errors in DeepSlice alignment. The Allen adult mouse brain coronal atlas was used to align all sections to ensure labeling of brain regions was consistent across ages (Figure S3). The manual alignment step allowed adjustment for any minor variations in section morphology across ages. Regions exhibiting tissue damage were excluded.

##### Fos+ cell detection

Images of the Fos channel were processed using a custom deep learning pipeline. This pipeline has largely the same architecture as our existing 3D pipeline, DeepCOUNT,^88^ but uses a 2D U-Net instead of a 3D U-Net. A network for segmenting Fos^+^ cells was trained and validated using manually annotated 100 × 100 pixel images. Following model training, each section was segmented for Fos^+^ cells. Cells were quantified using 2D maxima detection on the segmented images in FIJI. The number of detected cells in each region was divided by the area of that region to generate Fos density counts.

#### Dendritic spine quantification and classification

To quantify dendritic spine density on TRAPed cells, 3–5 dendritic segments were imaged in the RSP for each mouse for FIJI analysis. The number of spines were counted along secondary dendrites of TRAPed cells (2 dendrites per cell, 3–4 cells per mouse) to calculate spine density. Only neurons with their complete morphologies contained within the section were analyzed.

Dendritic spines were classified based on established morphological criteria.^69,70,94^ Analysis was conducted in ImageJ. z stack images were loaded, and spines were manually classified according to the following criteria. Stubby spines were defined as having a head length-to-width ratio of approximately 1, with no visible neck protrusion. Mushroom spines were characterized by large spine heads and narrow necks, with a length-to-width ratio greater than 1 and a head diameter greater than 0.5 μm. Thin spines were defined by small heads and elongated necks, with a length-to-width ratio of approximately 2, typical spine lengths between 1 and 3 μm, and head diameters less than 0.5 μm. Spines were only included in this analysis if they were in no way obscured by other structures, such as neighboring dendritic segments.

### QUANTIFICATION AND STATISTICAL ANALYSIS

Results were plotted and tested for statistical significance with MATLAB, R and GraphPad Prism v10. Statistical tests are described in the figure legends. Multiple comparisons with single variables were analyzed with one-way ANOVA with post-hoc Tukey’s test (comparing the mean of each column with that of every other column). For multiple comparisons with more than one variable, two-way ANOVA with post hoc Tukey’s or Sidak test was used. The relationships between TRAPed cell counts (normalized as learning indices) and group were analyzed using a behavior partial least-squares analysis^31,95,96^ implemented in R, resulting in two matrices for each age group containing contrasts and saliences respectively. Resampling statistics were used to determine the reliability of brain region saliences. The reliability of brain region saliences was determined using bootstrap ratios in which subjects retained their freezing levels but were resampled with replacement 1000 times. Original saliences were divided by the bootstrap-derived standard deviations to generate bootstrap ratios (saliences). Salience above 3 was considered reliable. No statistical methods were used to predetermine sample size. Sample sizes were determined based on similar published studies. In all figures, graphs show group means superimposed on individual data points from each animal.

## Supplementary Material

1

2

SUPPLEMENTAL INFORMATION

Supplemental information can be found online at https://doi.org/10.1016/j.celrep.2026.117768.

## Figures and Tables

**Figure 1.**
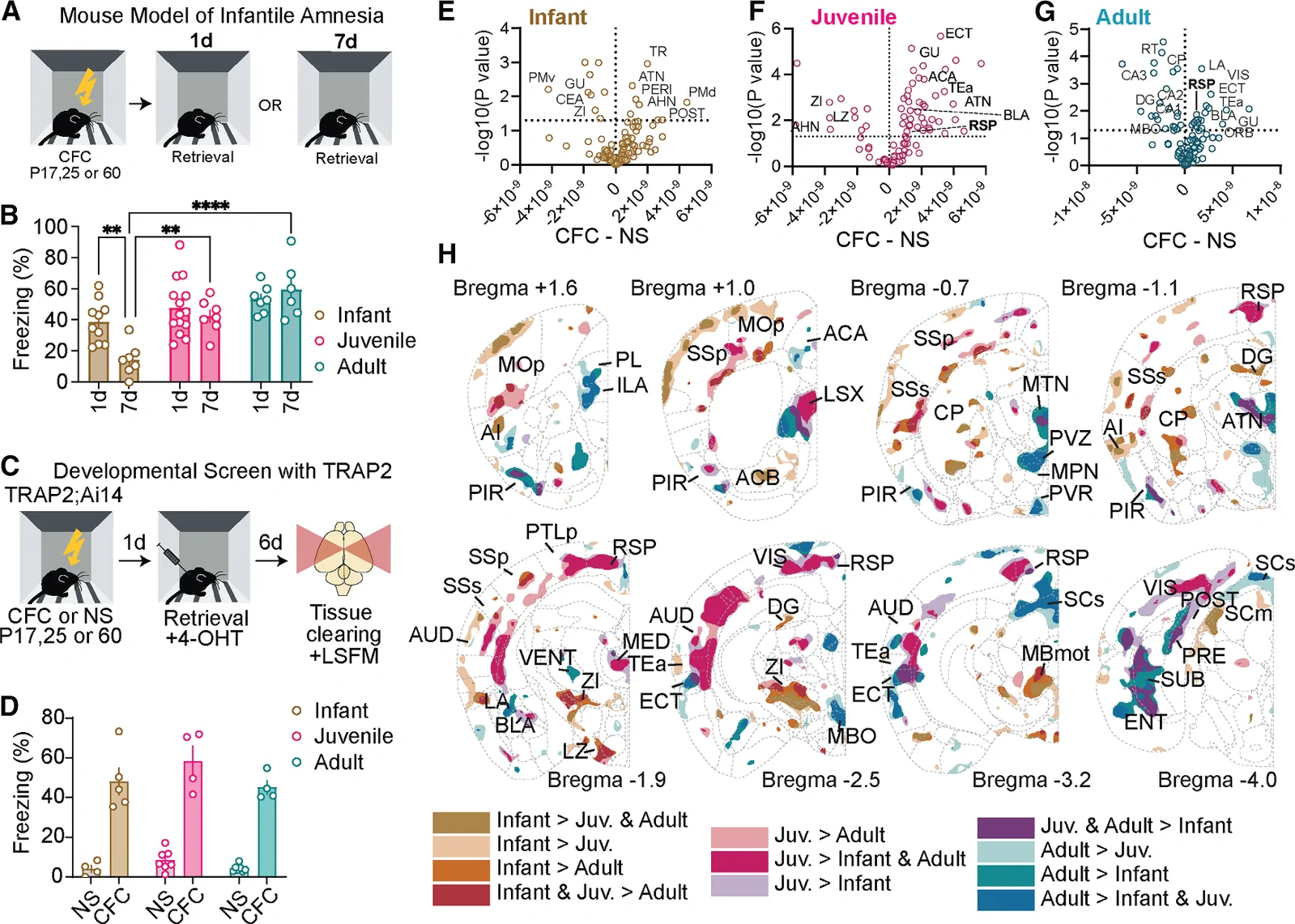
A developmental screen to identify neural signatures of infantile amnesia (A) Infant, juvenile, and adult mice underwent contextual fear conditioning and then memory testing 1 or 7 days later. (B) Percent time freezing for infant, juvenile, and adult wildtype mice during memory retrieval tests occurring 1 or 7 days after CFC. Two-way ANOVA with Tukey’s multiple comparisons test: F_age_(2,45) = 15.89, *p* < 0.0001, F_day_(1,45) = 3.56, *p* = 0.07, F_int_(2,45) = 4.21, *p* = 0.02, infant: *N* = 7 1 d, *N* = 10 7 d; juvenile: *N* = 14 1 d, *N* = 8 7 d; adult: *N* = 7 1 d, *N* = 6 7d. (C) Experimental design for a developmental screen. (D) Freezing behavior in TRAP mice used in the screen. Infant: *N* = 4 NS, *N* = 5 CFC; juvenile: *N* = 7 NS, *N* = 4 CFC; adult: *N* = 7 NS, *N* = 4 CFC. (E–G) Volcano plots of unpaired two-tailed t-tests comparing learning indices between CFC and NS mice at infant (E), juvenile (F), and adult (G) timepoints. Infant: *N* = 4 NS, *N* = 5 CFC; juvenile: *N* = 7 NS, *N* = 4 CFC; adult: *N* = 7 NS, *N* = 4 CFC. Horizontal dashed line corresponds to *p* < 0.05. (H) Voxel-based analysis compares learning indices in infant, juvenile, and adult CFC brains. Values from each voxel underwent one-way ANOVA with Tukey’s multiple comparisons test (Infant: *N* = 5; Juvenile: *N* = 4; Adult: *N* = 4). Colored voxels indicate *p* < 0.05 in Tukey’s test for the comparisons denoted in the legend. See Tables S1 and S2 for abbreviations and statistics. Data are presented as mean ± SEM. **p < 0.01 and ****p < 0.0001.

**Figure 2.**
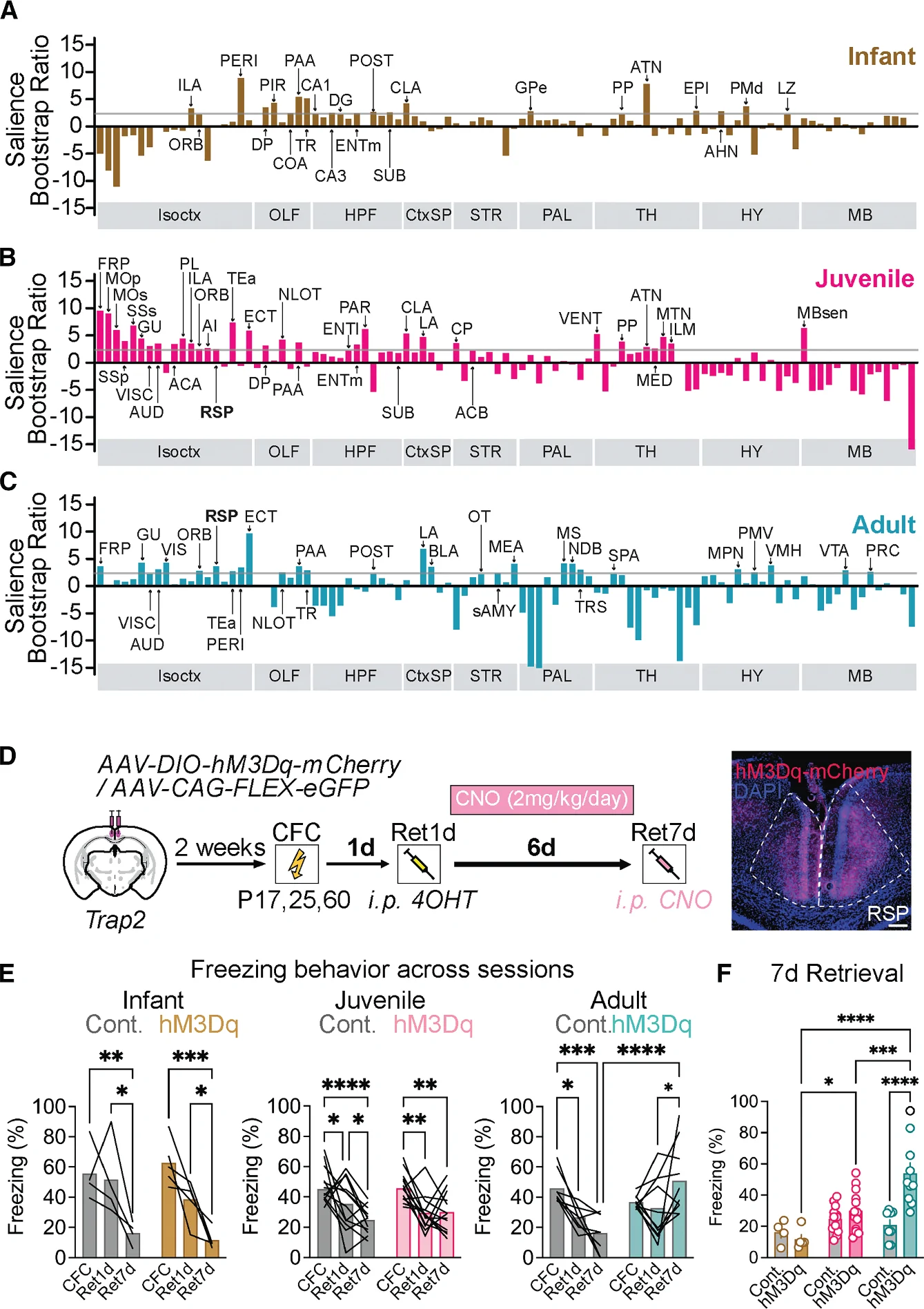
Chemogenetic reactivation of TRAPed RSP neurons increases freezing during memory retrieval in an age-dependent manner (A–C) Task PLS analysis of regional learning indices in infant (A), juvenile (B), and adult (C) mice. Gray line indicates threshold value of 2. (D) Experimental design for chemogenetic activation of RSP ensembles. (E) Percent freezing levels across behavior sessions in control (gray) and hM3Dq (color) groups for infants, juveniles, and adults. Two-way ANOVA with Sidak multiple comparisons test: Infant: F_day_(2,14) = 20.01, *p* < 0.0001; F_virus_(1,7) = 0.338, *p* = 0.579; F_int_(2,14) = 0.947, *p* = 0.411; *N* = 4 Cont, *N* = 5 hM3dq; juvenile: F_day_(2,46) = 16.87, *p* < 0.0001; F_virus_(1,23) = 0.053, *p* = 0.82; F_int_(2,46) = 1.223, *p* = 0.31; *N* = 12 Cont, *N* = 13 hM3dq; adult: F_day_(2,32) = 3.22, *p* = 0.05; F_virus_(1,16) = 4.51, *p* = 0.04; F_int_(2,32) = 11.5, *p* = 0.002; *N* = 8 Cont, *N* = 10 hM3dq. (F) Comparing freezing during 7-day retrieval across ages. two-way ANOVA with Sidak multiple comparisons test: F_age_(2,46) = 9.64, *p* = 0.0003; F_virus_(1,46) = 8.03, *p* = 0.007; F_int_(2,46) = 8.37, *p* = 0.0008. Data are presented as mean ± SEM. *p < 0.05, **p < 0.01, ***p < 0.001, and ****p < 0.0001. Scale bars, 100 μm.

**Figure 3.**
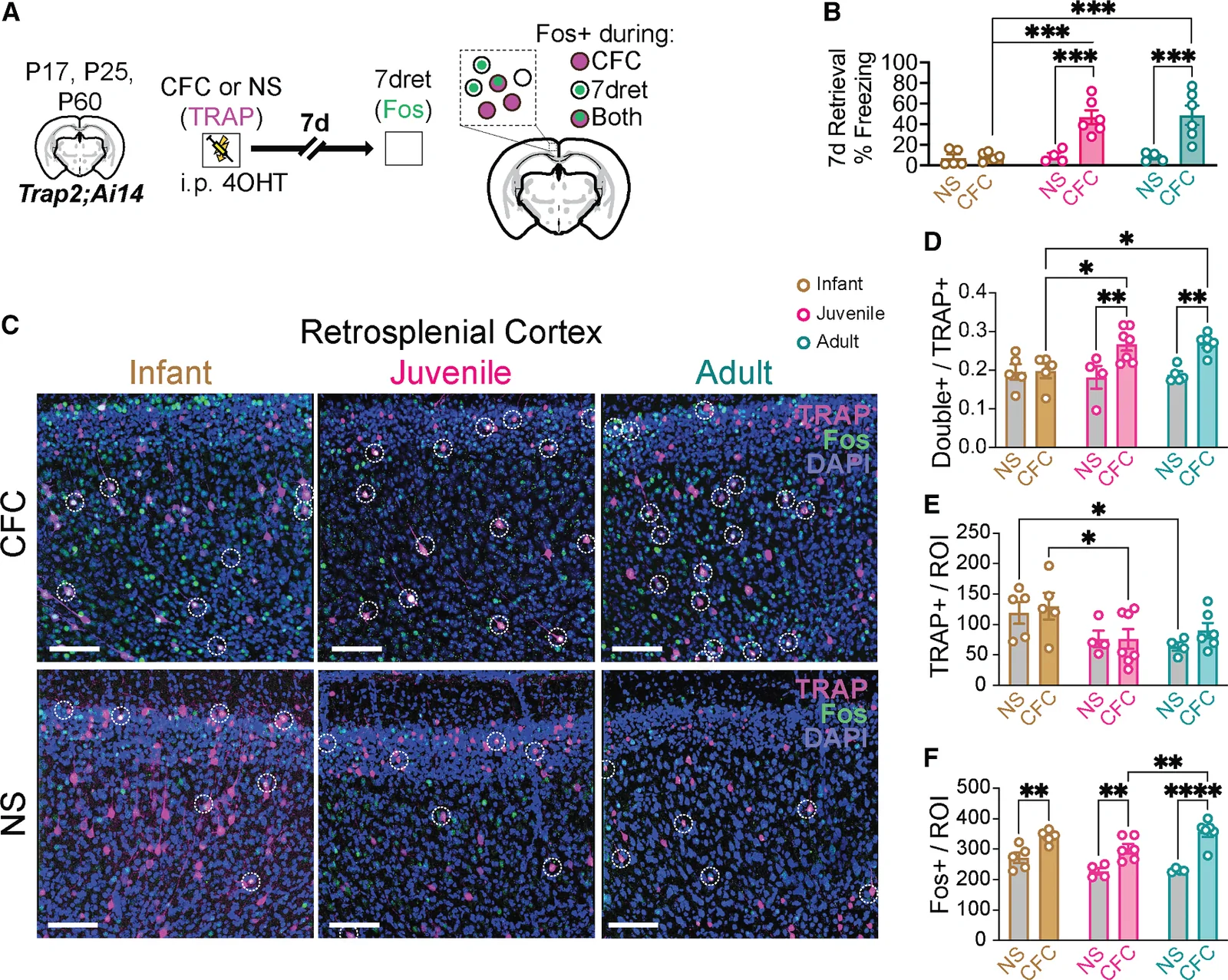
Increased reactivation of RSP memory ensembles in juveniles and adults (A) Experiment design for TRAPing at CFC/NS and Fos staining following 7-day retrieval. (B) Freezing behavior during 7-day retrieval. Two-way ANOVA with Sidak multiple comparisons test: F_shock_(1,24) = 27.5, *p* < 0.0001, F_age_(2,24) = 7.14, *p* = 0.004; F_int_(2,24) = 6.27, *p* = 0.0064; infant: *N* = 5 CFC, *N* = 5 NS; juvenile: *N* = 6 CFC, *N* = 4 NS; adult: *N* = 6 CFC, *N* = 5 NS. (C) Representative examples of TRAPed (magenta), Fos+ cells (green), and double-labeled reactivated cells (white) in RSP after CFC (top) or in NS control mice (bottom). (D) Reactivated TRAPed RSP neurons after 7 d retrieval. Two-way ANOVA with Tukey’s multiple comparisons test: F_shock_(1,26) = 15.19, *p* = 0.0006, F_age_(2,26) = 1.90, *p* = 0.17; F_int_(2,26) = 3.5, *p* = 0.04. Infant: *N* = 5 CFC, *N* = 5 NS; juvenile: *N* = 7 CFC, *N* = 4 NS; Adult: *N* = 6 CFC, *N* = 5 NS. (E) TRAPed RSP cells. Two-way ANOVA with Tukey’s multiple comparisons test: F_shock_(1,25) = 0.84, *p* = 0.37, F_age_(2,25) = 5.53, *p* = 0.01; F_int_(2,25) = 0.31, *p* = 0.74. Infant: *N* = 5 CFC, *N* = 5 NS; Juvenile: *N* = 7 CFC, *N* = 4 NS; Adult: *N* = 6 CFC, *N* = 4 NS. (F) Fos+ RSP cells in RSP after 7 d retrieval. two-way ANOVA with Tukey’s multiple comparisons test: F_shock_(1,24) = 57.53, *p* < 0.0001, F_age_(2,24) = 4.31, *p* = 0.02; F_int_(2,24) = 2.38, *p* = 0.11. Infant: *N* = 5 CFC, *N* = 5 NS; Juvenile: *N* = 7 CFC, *N* = 4 NS; Adult: *N* = 6 CFC, *N* = 4 NS. Data are presented as mean ± SEM. *p < 0.05, **p < 0.01, ***p < 0.001, and ****p < 0.0001. Scale bars, 100 μm.

**Figure 4.**
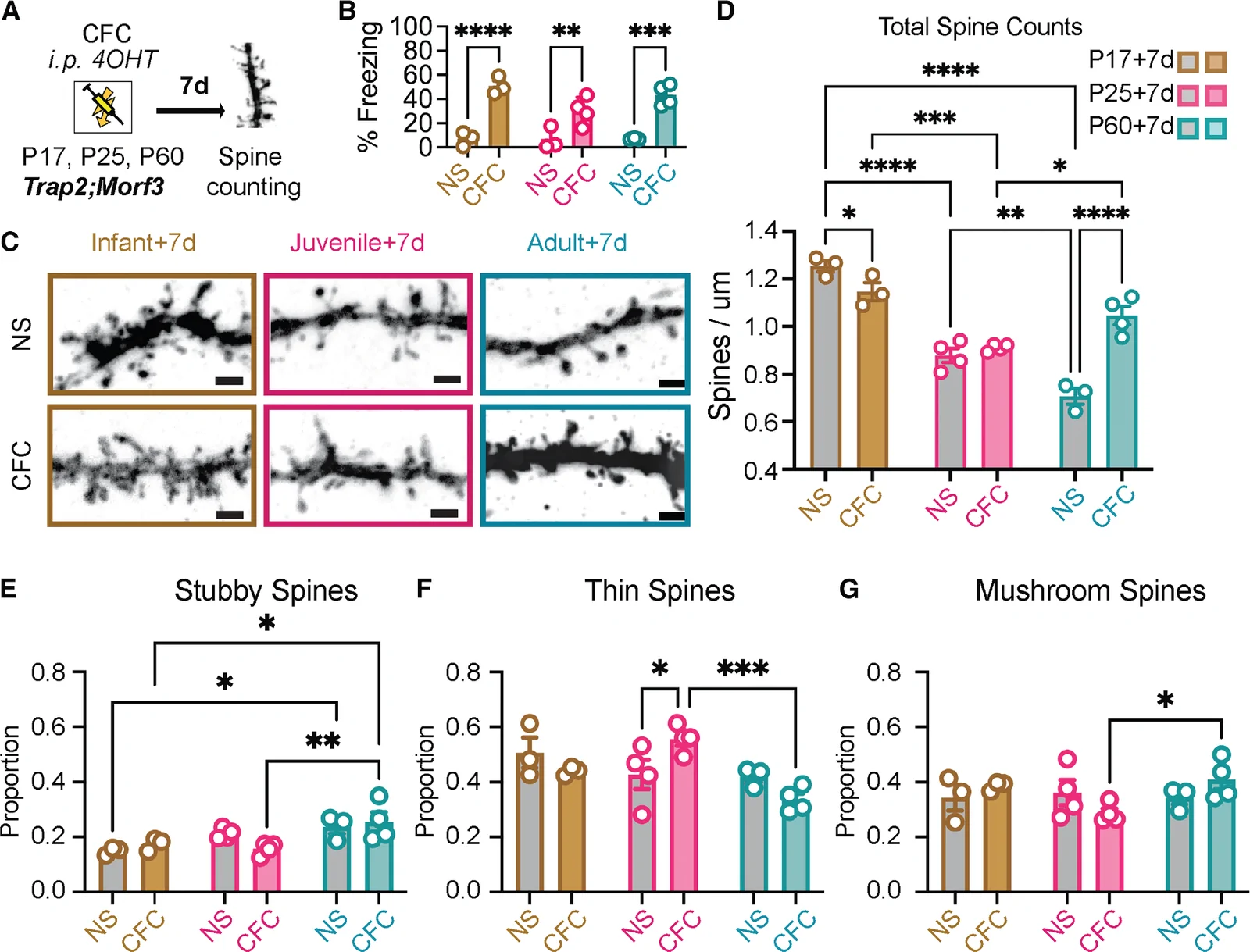
Developmental spine pruning and structural plasticity in RSP neurons (A) Experiment design for analyzing dendritic spines on TRAPed RSP neurons. (B) Freezing during CFC or NS context exposure. Two-way ANOVA with Tukey’s multiple comparisons test: F_group_(1,14) = 82.93, *p* < 0.0001, F_age_(2,14) = 2.65, *p* = 0.11, F_int_(2,14) = 2.50, *p* = 0.12. (C) Representative examples of CFC- or NS-TRAPed RSP spines in infants, juveniles, or adults. Scale bars, 2 μm. (D) Spine density in RSP TRAPed cells. Two-way ANOVA with Tukey’s multiple comparisons test: F_group_(1,15) = 12.54, *p* = 0.003, F_age_(2,15) = 67.02, *p* < 0.0001, F_int_(2,15) = 27.21, *p* < 0.0001. (E) Proportion of stubby spines across ages. Two-way ANOVA with Tukey’s multiple comparisons test: F_group_(1,16) = 0.594, *p* = 0.452, F_age_(2,16) = 3.82, *p* = 0.044, F_int_(2,16) = 1.03, *p* = 0.37. (F) Same as (E) for thin spines. Two-way ANOVA with Tukey’s multiple comparisons test: F_group_(1,15) = 0.06, *p* = 0.81, F_age_(2,15) = 5.75, *p* = 0.014, F_int_(2,15) = 5.01, *p* = 0.052. (G) Same as (E) for mushroom spines. Two-way ANOVA with Tukey’s multiple comparisons test: F_group_(1,15) = 0.06, *p* = 0.81, F_age_(2,16) = 1.34, *p* = 0.289, F_int_(2,16) = 2.85, *p* = 0.086. Infant: *N* = 3 CFC, *N* = 3 NS mice; Juvenile: *N* = 4 CFC, *N* = 4 NS mice; Adult: *N* = 4 CFC, *N* = 3 NS mice; 6–8 dendritic segments analyzed per mouse. Data are presented as mean ± SEM. *p < 0.05, **p < 0.01, ***p < 0.001, and ****p < 0.0001.

**Figure 5.**
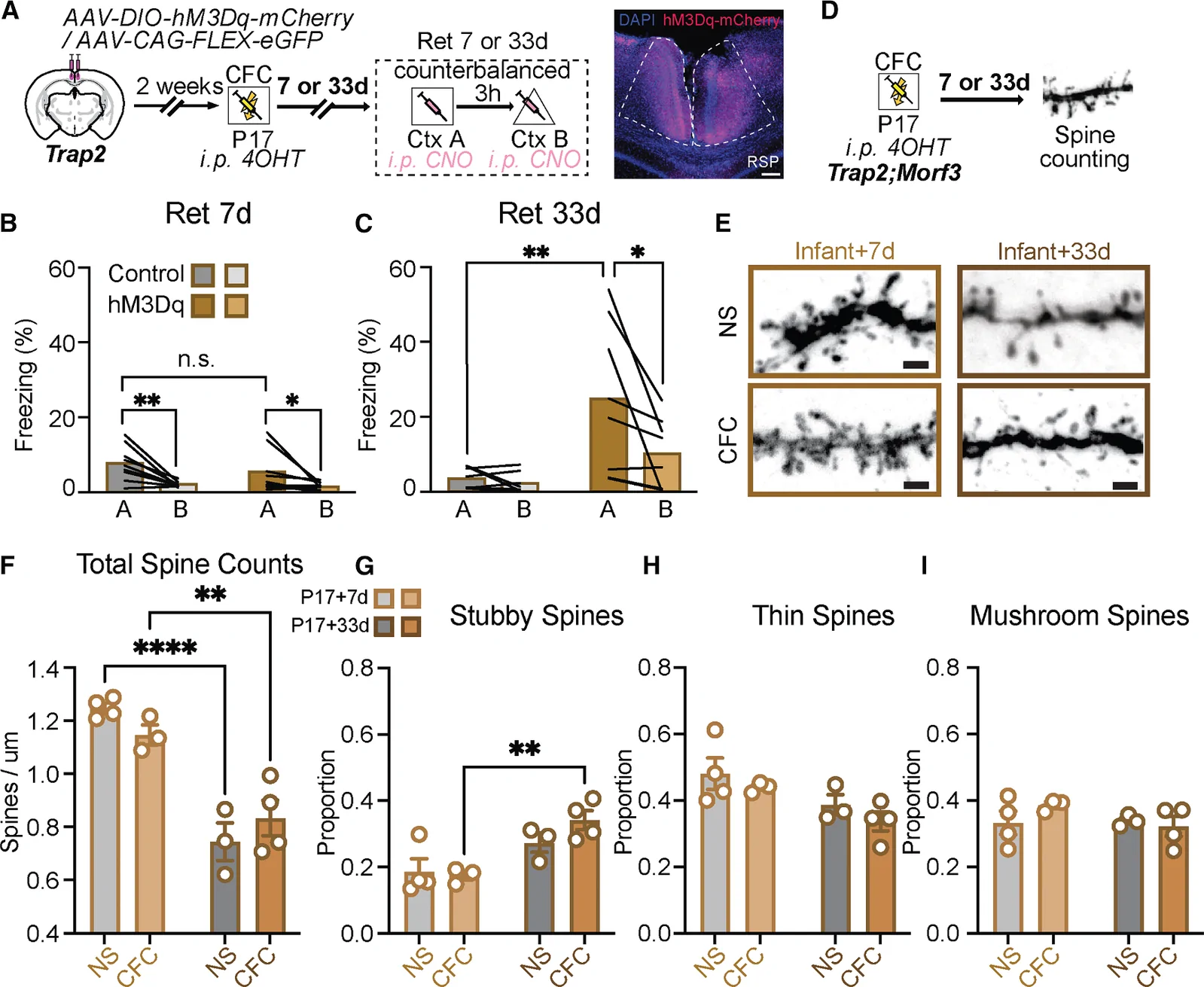
Chemogenetic reactivation of learning encoding ensembles in RSP 33 d after training recovers infant memories (A) Experimental design. Scale bars, 100 μm. (B) Percent freezing levels in contexts A and B during the 7-day retrieval tests in control (gray) and hM3Dq (brown) mice. Two-way ANOVA with Holm-Sidak multiple comparisons test: F_virus_(1,16) = 1.226, *p* = 0.2845; F_context_(1,16) = 16.65, *p* = 0.0009; F_int_(1,16) = 0.5968, *p* = 0.4511. Cont: *N* = 9, hM3Dq: *N* = 9. (C) Same as B but for a 33-day retrieval. Two-way ANOVA with Holm-Sidak multiple comparisons test: F_virus_(1,14) = 9.062, *p* = 0.0094; F_context_(1,14) = 7.41, *p* = 0.0165; F_int_(1,14) = 5.234, *p* = 0.0382. Cont: *N* = 8, hM3Dq: *N* = 8. (D) Experiment design for dendritic spine analysis of CFC- or NS-TRAPed RSP neurons. (E) Representative examples of CFC- or NS-TRAPed RSP spines in infants+7 d 33 d. Infant+7 d image is identical to that depicted in Figure 4. Scale bars, 2 μm. (F) Quantification of spine density in P17 TRAPed RSP cells. Two-way ANOVA with Sidak multiple comparisons test: F_group_(1,10) = 0.01, *p* = 0.92; F_age_(1,10) = 59.39, *p* < 0.0001; F_int_(1,10) = 3.22, *p* = 0.10. +7 d: Cont: *N* = 4, CFC: *N* = 3; +33 d: Cont: *N* = 3, CFC: *N* = 4. (G) Proportion of stubby spines across ages. Two-way ANOVA with Sidak multiple comparisons test: F_group_(1,10) = 0.78, *p* = 0.39, F_age_(1,10) = 16.59, *p* = 0.0022, F_int_(1,10) = 1.65, *p* = 0.23. (H) Same as (G) for thin spines. Two-way ANOVA with Sidak multiple comparisons test: F_group_(1,10) = 1.67, *p* = 0.23, F_age_(1,10) = 7.68, *p* = 0.0198, F_int_(1,10) = 0.02, *p* = 0.89. (I) Same as (G) for mushroom spines. Two-way ANOVA with Sidak multiple comparisons test: F_group_(1,10) = 0.41, *p* = 0.54, F_age_(1,10) = 1.06, *p* = 0.33, F_int_(1,10) = 1.56, *p* = 0.24. Data are presented as mean ± SEM. *p < 0.05, **p < 0.01, and ****p < 0.0001. Infant + 7 d data are identical to that depicted in Figure 4.

**Table 1.** List of abbreviations

ACA	anterior cingulate cortex
ACB	nucleus accumbens
AHN	anterior hypothalamic nucleus
AI	agranular insular area
ATN	anterior group of the dorsal thalamus
AUD	auditory areas
APN	anterior pretectal nucleus
BLA	basolateral amygdala
BMA	basomedial amygdala
BST	bed nucleus of the stria terminalis
CA1	hippocampal field CA1
CA2	hippocampal field CA2
CA3	hippocampal field CA3
CEA	central amygdalar nucleus
CLA	claustrum
CNU	cerebral nuclei
COA	cortical amygdalar area
CP	caudoputamen
CTXsp	cortical subplate
DG	dentate gyrus
DP	dorsal peduncular area
ECT	ectorhinal cortex
ENTl	entorhinal area, lateral part
ENTm	entorhinal area, medial part
EP	endopiriform nucleus
EPI	epithalamus
FRP	frontal pole, cerebral cortex
GENd	geniculate group, dorsal thalamus
GENv	geniculate group, ventral thalamus
GPe	globus pallidus, external segment
GPi	globus pallidus, internal segment
GU	gustatory areas
HB	hindbrain
HPF	hippocampal formation
IC	inferior colliculus
IGL	intergeniculate leaflet of the lateral geniculate complex
IL	infralimbic cortex
ILM	intralaminar nucleus of the dorsal thalamus
Isoctx	isocortex
LA	lateral amygdalar nucleus
LAT	lateral group of the dorsal thalamus
LSX	lateral septal complex
LZ	hypothalamic lateral zone
MA	magnocellular nucleus
MB	midbrain
MBO	mammillary body
MBmot	midbrain, motor related
MBsen	midbrain, sensory related
MEA	medial amygdala
MED	medial group of the dorsal thalamus
MOp	primary motor area
MOs	secondary motor area
mPFC	medial prefrontal cortex
MPN	medial preoptic nucleus
MPT	medial pretectal area
MS	medial septal nucleus
MTN	midline group of the dorsal thalamus
NDB	nucleus of the diagonal band
NLOT	nucleus of the lateral olfactory tract
OLF	olfactory areas
ORB	orbital area
OT	olfactory tubercle
PA	posterior amygdalar nucleus
PAA	piriform-amygdalar area
PAG	periaqueductal gray
PAL	pallidum
PAR	parasubiculum
PERI	perirhinal area
PH	posterior hypothalamic nucleus
PIR	piriform area
PL	prelimbic area
PMd	dorsal premammilary nucleus
PMv	ventral premammilary nucleus
POST	postsubiculum
PP	peripeduncular nucleus
PPN	pedunculopontine nucleus
PRC	precommisural nucleus
PRE	presubiculum
ProS	prosubiculum
PTLp	posterior parietal association areas
PVHd	paraventricular hypothalamic nucleus, descending division PVR periventricular region
PVZ	periventricular zone
RAmb	midbrain raphe nuclei
RSP	retrosplenial cortex
RT	reticular nucleus of the thalamus
sAMY	striatum-like amygdala
SCm	superior colliculus, motor related
SCs	superior colliculus, sensory related
SI	substantia innominata
SNc	substantia nigra, compact part
SNr	substantia nigra, reticular part
SPA	subparafascicular area
SPF	subparafascicular nucleus
SSp	primary somatosensory area
SSs	secondary somatosensory area
STRv	striatum ventral region
SUB	subiculum
TEa	temporal association area
TH	thalamus
TR	postpiriform transition area
TT	taenia tecta
VENT	ventral group of the dorsal thalamus
VIS	visual areas
VMH	ventromedial hypothalamic nucleus
VTA	ventral tegmental area
ZI	zona incerta

**KEY RESOURCES TABLE:** 

REAGENT or RESOURCE	SOURCE	IDENTIFIER
Antibodies		
Rabbit anti-Fos	Synaptic systems	RRID:AB_2891278
Chicken anti-GFP	Aves Labs, Cat #1020	RRID:AB_10000240
Rat anti-mCherry	Thermo Fisher	RRID: AB_2536611; Cat# M11 217
Rabbit anti-RFP	Rockland	RRID: AB_2209751; Cat# 601-401-379
Rabbit anti-V5	Bethyl	RRID: AB_67586; Cat# A190-120A
Bacterial and virus strains		
AAV8-hSyn-DIO-hM3D(Gq)-mCherry	Addgene	44361
AAV1-pCAG-FLEX-eGFP-WPRE	Addgene	51502
AAV5-Ef1a-DIO-mscarlet	Addgene	131002-AAV5
AAV-DJ-CMV-DIO-EGFP-2A-TeNT	Stanford Gene Vector and Virus Core	GVV-AAV071
AAV8-hsyn-DIO-hM4Di-mCherry	Addgene	44362-AAV8
AAV8-hsyn-hM4Di-mCherry	Addgene	50475-AAV8
Chemicals, peptides, and recombinant proteins		
Clozapine-N-Oxide	Fisher	63-291-0
4-hydroxytamoxifen	Sigma	H6278
Phosphate Buffered Saline	Ambion	AM9624
Triton X-100	Sigma	×100-500ML
Tween 20	Sigma	P9416
DMSO	Fisher	D128-4
Sodium Azide	Sigma	58032-100G
Donkey Serum	Jackson Immunoresearch	017-000-121
Glycine	Sigma	G7126-500G
Heparin	Sigma	H3393-50KU
Methanol	Fisher	A412SK-4
Hydrogen Peroxide 30%	Sigma	216763-100ML
Dicholoromethane	Sigma	270997-12 × 100ML
Dibenzyl Ether	Sigma	108014-1KG
Paraformaldehyde 16%	EMS	15710-S
Deposited data		
Whole brain TRAP quantification	This paper	
Experimental models: Organisms/strains		
Mouse C57/Bl6J	Jackson Laboratory	JAX:000664
TRAP2	Jackson Laboratory	JAX:030323
Ai14	Jackson Laboratory	JAX:007914
Software and algorithms		
TRAILMAP	Friedmann et al.^75^	https://doi.org/10.1073/pnas.1918465117
DeepCOUNT	Gongwer et al.^18^	https://doi.org/10.1523/JNEUROSCI.0697-23.2023; https://doi.org/10.5281/zenodo.21283955.
FIJI ImageJ (v2)	Schindelin et al.^80^	SCR_003070
Python (v3)	Anaconda	https://conda.io
Adobe Illustrator	Adobe	SCR_010279
GraphPad PRISM (v10)	GraphPad	SCR_002798
MATLAB	Mathworks	SCR_001622
Arduino	Arduino	https://www.arduino.cc/
BehaviorDEPOT	Gabriel et al.^77^	https://doi.org/10.7554/eLife.74314; https://doi.org/10.5281/zenodo.21283773.
DeepLabCut	Mathis et al.^76^	https://doi.org/10.1038/s41596-019-0176-0
ABBA		https://doi.org/10.1016/j.celrep.2025.115876
DeepSlice	Carey et al.^79^	https://doi.org/10.1038/s41467-023-41645-4

## Highlights

- Brain-wide screen highlights RSP maturation during transitions in long-term memory
- Lasting memory coincides with developmental increases in RSP ensemble reactivation
- RSP ensemble activation reinstates infant memories after a developmental delay
- Developmental spine remodeling mirrors RSP ensemble-mediated memory recall

## References

[1] Bauer PJ, Tasdemir-Ozdes A, and Larkina M (2014). Adults’ reports of their earliest memories: Consistency in events, ages, and narrative characteristics over time. Conscious. Cognit. 27, 76–88. 10.1016/j.concog.2014.04.008. PMC4104227 24836979

[2] Campbell BA, and Campbell EH (1962). Retention and extinction of learned fear in infant and adult rats. J. Comp. Physiol. Psychol. 55, 1–8. 10.1037/h0049182. 13876002

[3] Bauer PJ (2015). Development of episodic and autobiographical memory: The importance of remembering forgetting. Dev. Rev. 38, 146–166. 10.1016/j.dr.2015.07.011. PMC4669902 26644633

[4] Lloyd ME, and Newcombe NS (2009). Implicit memory in childhood: Reassessing developmental invariance. In The Development of Memory in Infancy and Childhood, 93, Second Edition (Psychology Press), p. 113.

[5] Ramsaran AI, Schlichting ML, and Frankland PW (2019). The ontogeny of memory persistence and specificity. Dev. Cogn. Neurosci. 36, 100591. 10.1016/j.dcn.2018.09.002. PMC6969236 30316637

[6] Campbell BA, and Spear NE (1972). Ontogeny of memory. Psychol. Rev. 79, 215–236. 10.1037/h0032690. 4341445

[7] Callaghan B, Meyer H, Opendak M, Van Tieghem M, Harmon C, Li A, Lee FS, Sullivan RM, and Tottenham N (2019). Using a Developmental Ecology Framework to Align Fear Neurobiology Across Species. Annu. Rev. Clin. Psychol. 15, 345–369. 10.1146/annurev-clinpsy-050718-095727. PMC7219957 30786246

[8] Akers KG, Arruda-Carvalho M, Josselyn SA, and Frankland PW (2012). Ontogeny of contextual fear memory formation, specificity, and persistence in mice. Learn. Mem. 19, 598–604. 10.1101/lm.027581.112. 23161449

[9] Akers KG, Martinez-Canabal A, Restivo L, Yiu AP, De Cristofaro A, Hsiang HLL, Wheeler AL, Guskjolen A, Niibori Y, Shoji H, (2014). Hippocampal neurogenesis regulates forgetting during adulthood and infancy. Science 344, 598–602. 10.1126/science.1248903. 24812394

[10] Guskjolen A, Kenney JW, de la Parra J, Yeung BA, Josselyn SA, and Frankland PW (2018). Recovery of “Lost” Infant Memories in Mice. Curr. Biol. 28, 2283–2290.e3. 10.1016/j.cub.2018.05.059. 29983316

[11] Guskjolen A, Josselyn SA, and Frankland PW (2017). Age-dependent changes in spatial memory retention and flexibility in mice. Neurobiol. Learn. Mem. 143, 59–66. 10.1016/j.nlm.2016.12.006. 27988313

[12] Li S, Kim JH, and Richardson R (2012). Updating memories: Changing the involvement of the prelimbic cortex in the expression of an infant fear memory. Neuroscience 222, 316–325. 10.1016/j.neuroscience.2012.06.038. 22735578

[13] Travaglia A, Bisaz R, Sweet ES, Blitzer RD, and Alberini CM (2016). Infantile amnesia reflects a developmental critical period for hippocampal learning. Nat. Neurosci. 19, 1225–1233. 10.1038/nn.4348. PMC5003643 27428652

[14] Alberini CM, and Travaglia A (2017). Infantile Amnesia: A Critical Period of Learning to Learn and Remember. J. Neurosci. 37, 5783–5795. 10.1523/jneurosci.0324-17.2017. PMC5473198 28615475

[15] Kim JH, Li S, Hamlin AS, McNally GP, and Richardson R (2012). Phosphorylation of mitogen-activated protein kinase in the medial prefrontal cortex and the amygdala following memory retrieval or forgetting in developing rats. Neurobiol. Learn. Mem. 97, 59–68. 10.1016/j.nlm.2011.09.005. 21963362

[16] Spear NE, and Parsons PJ (1976). Analysis of a Reactivation Treatment: Ontogenetic Determinants of Alleviated Forgetting. In Processes of Animal Memory (PLE: Memory) (Psychology Press), pp. 135–165.

[17] Campbell BA, and Jaynes J (2000). Reinstatement. 20 (Lawrence Erlbaum Associates Publishers).

[18] Callaghan BL, and Richardson R (2011). Maternal separation results in early emergence of adult-like fear and extinction learning in infant rats. Behav. Neurosci. 125, 20–28. 10.1037/a0022008. 21319883

[19] Bath KG, Manzano-Nieves G, and Goodwill H (2016). Early life stress accelerates behavioral and neural maturation of the hippocampus in male mice. Horm. Behav. 82, 64–71. 10.1016/j.yhbeh.2016.04.010. PMC5308418 27155103

[20] Callaghan BL, and Tottenham N (2016). The Stress Acceleration Hypothesis: Effects of early-life adversity on emotion circuits and behavior. Curr. Opin. Behav. Sci. 7, 76–81. 10.1016/j.cobeha.2015.11.018. PMC5890821 29644262

[21] Frankland PW, and Bontempi B (2005). The organization of recent and remote memories. Nat. Rev. Neurosci. 6, 119–130. 10.1038/nrn1607. 15685217

[22] Tonegawa S, Morrissey MD, and Kitamura T (2018). The role of engram cells in the systems consolidation of memory. Nat. Rev. Neurosci. 19, 485–498. 10.1038/s41583-018-0031-2. 29970909

[23] Frankland PW, and Bontempi B (2006). Fast track to the medial prefrontal cortex. Proc. Natl. Acad. Sci. 103, 509–510. 10.1073/pnas.0510133103. PMC1334679 16407121

[24] Ramsaran AI, Wang Y, Golbabaei A, Aleshin S, de Snoo ML, Yeung B.r.A., Rashid AJ, Awasthi A, Lau J, Tran LM, (2023). A shift in the mechanisms controlling hippocampal engram formation during brain maturation. Science 380, 543–551. 10.1126/science.ade6530. 37141366

[25] Klune CB, Jin B, and Denardo LA (2021). Linking mPFC circuit maturation to the developmental regulation of emotional memory and cognitive flexibility. eLife 10, e64567. 10.7554/elife.64567. PMC8099425 33949949

[26] Sugar J, and Witter MP (2016). Postnatal development of retrosplenial projections to the parahippocampal region of the rat. eLife 5, e13925. 10.7554/elife.13925. PMC4859804 27008178

[27] Haugland KG, Sugar J, and Witter MP (2019). Development and topographical organization of projections from the hippocampus and parahippocampus to the retrosplenial cortex. Eur. J. Neurosci. 50, 1799–1819. 10.1111/ejn.14395. PMC6767700 30803071

[28] DeNardo LA, Liu CD, Allen WE, Adams EL, Friedmann D, Fu L, Guenthner CJ, Tessier-Lavigne M, and Luo L (2019). Temporal evolution of cortical ensembles promoting remote memory retrieval. Nat. Neurosci. 22, 460–469. 10.1038/s41593-018-0318-7. PMC6387639 30692687

[29] Allen WE, DeNardo LA, Chen MZ, Liu CD, Loh KM, Fenno LE, Ramakrishnan C, Deisseroth K, and Luo L (2017). Thirst-associated preoptic neurons encode an aversive motivational drive. Science 357, 1149–1155. 10.1126/science.aan6747. PMC5723384 28912243

[30] Madisen L, Zwingman TA, Sunkin SM, Oh SW, Zariwala HA, Gu H, Ng LL, Palmiter RD, Hawrylycz MJ, Jones AR, (2010). A robust and high-throughput Cre reporting and characterization system for the whole mouse brain. Nat. Neurosci. 13, 133–140. 10.1038/nn.2467. PMC2840225 20023653

[31] Wheeler AL, Teixeira CM, Wang AH, Xiong X, Kovacevic N, Lerch JP, McIntosh AR, Parkinson J, and Frankland PW (2013). Identification of a Functional Connectome for Long-Term Fear Memory in Mice. PLoS Comput. Biol. 9, e1002853. 10.1371/journal.pcbi.1002853. PMC3536620 23300432

[32] Kwapis JL, Jarome TJ, Lee JL, and Helmstetter FJ (2015). The retrosplenial cortex is involved in the formation of memory for context and trace fear conditioning. Neurobiol. Learn. Mem. 123, 110–116. 10.1016/j.nlm.2015.06.007. PMC4754129 26079095

[33] Trask S, Ferrara NC, Jasnow AM, and Kwapis JL (2021). Contributions of the rodent cingulate-retrosplenial cortical axis to associative learning and memory: A proposed circuit for persistent memory maintenance. Neurosci. Biobehav. Rev. 130, 178–184. 10.1016/j.neubiorev.2021.08.023. PMC8511298 34450181

[34] De Sousa AF, Cowansage KK, Zutshi I, Cardozo LM, Yoo EJ, Leutgeb S, and Mayford M (2019). Optogenetic reactivation of memory ensembles in the retrosplenial cortex induces systems consolidation. Proc. Natl. Acad. Sci. USA 116, 8576–8581. 10.1073/pnas.1818432116. PMC6486739 30877252

[35] Keene CS, and Bucci DJ (2008). Contributions of the retrosplenial and posterior parietal cortices to cue-specific and contextual fear conditioning. Behav. Neurosci. 122, 89–97. 10.1037/0735-7044.122.1.89 18298252

[36] Frankland PW, Josselyn SA, and Köhler S (2019). The neurobiological foundation of memory retrieval. Nat. Neurosci. 22, 1576–1585. 10.1038/s41593-019-0493-1. PMC6903648 31551594

[37] Cowansage KK, Shuman T, Dillingham B, Chang A, Golshani P, and Mayford M (2014). Direct reactivation of a coherent neocortical memory of context. Neuron 84, 432–441. 10.1016/j.neuron.2014.09.022. PMC4372249 25308330

[38] Pedrosa R, Nazari M, Kergoat L, Bernard C, Mohajerani M, Stella F, and Battaglia F (2024). Hippocampal ripples coincide with “up-state” and spindles in retrosplenial cortex. Cerebr. Cortex 34, bhae083. 10.1093/cercor/bhae083. 38494417

[39] Karimi Abadchi J, Nazari-Ahangarkolaee M, Gattas S, Bermudez-Contreras E, Luczak A, McNaughton BL, and Mohajerani MH (2020). Spatiotemporal patterns of neocortical activity around hippocampal sharp-wave ripples. eLife 9, e51972. 10.7554/elife.51972. PMC7096182 32167467

[40] Kaplan R, Adhikari M, Hindriks R, Mantini D, Murayama Y, Logothetis N, and Deco G (2016). Hippocampal Sharp-Wave Ripples Influence Selective Activation of the Default Mode Network. Curr. Biol. 26, 686–691. 10.1016/j.cub.2016.01.017. PMC4791429 26898464

[41] Chen Z, and Wilson MA (2017). Deciphering Neural Codes of Memory during Sleep. Trends Neurosci. 40, 260–275. 10.1016/j.tins.2017.03.005. PMC5434457 28390699

[42] Girardeau G, Benchenane K, Wiener SI, Buzsáki G, and Zugaro MB (2009). Selective suppression of hippocampal ripples impairs spatial memory. Nat. Neurosci. 12, 1222–1223. 10.1038/nn.2384. 19749750

[43] Wilson MA, and McNaughton BL (1994). Reactivation of hippocampal ensemble memories during sleep. Science 265, 676–679. 10.1126/science.8036517. 8036517

[44] Wang L, Park L, Wu W, King D, Vega-Medina A, Raven F, Martinez J, Ensing A, McDonald K, Yang Z, (2024). Sleep-dependent engram reactivation during hippocampal memory consolidation associated with subregion-specific biosynthetic changes. iScience 27, 109408. 10.1016/j.isci.2024.109408. PMC10957462 38523798

[45] Tomé DF, Zhang Y, Aida T, Mosto O, Lu Y, Chen M, Sadeh S, Roy DS, and Clopath C (2024). Dynamic and selective engrams emerge with memory consolidation. Nat. Neurosci. 27, 561–572. 10.1038/s41593-023-01551-w. PMC10917686 38243089

[46] Goto A, Bota A, Miya K, Wang J, Tsukamoto S, Jiang X, Hirai D, Murayama M, Matsuda T, McHugh TJ, (2021). Stepwise synaptic plasticity events drive the early phase of memory consolidation. Science 374, 857–863. 10.1126/science.abj9195. 34762472

[47] Armbruster BN, Li X, Pausch MH, Herlitze S, and Roth BL (2007). Evolving the lock to fit the key to create a family of G protein-coupled receptors potently activated by an inert ligand. Proc. Natl. Acad. Sci. USA 104, 5163–5168. 10.1073/pnas.0700293104. PMC1829280 17360345

[48] Tsai T-C, Yu T-H, Hung Y-C, Fong L-I, and Hsu K-S (2022). Distinct Contribution of Granular and Agranular Subdivisions of the Retrosplenial Cortex to Remote Contextual Fear Memory Retrieval. J. Neurosci. 42, 877–893. 10.1523/jneurosci.1303-21.2021. PMC8808736 34876468

[49] Corcoran KA, Frick BJ, Radulovic J, and Kay LM (2016). Analysis of coherent activity between retrosplenial cortex, hippocampus, thalamus, and anterior cingulate cortex during retrieval of recent and remote context fear memory. Neurobiol. Learn. Mem. 127, 93–101. 10.1016/j.nlm.2015.11.019. PMC4718831 26691782

[50] Sugar J, Witter MP, van Strien NM, and Cappaert NLM (2011). The Retrosplenial Cortex: Intrinsic Connectivity and Connections with the (Para)Hippocampal Region in the Rat. An Interactive Connectome. Front. Neuroinf. 5. 10.3389/fninf.2011.00007. PMC3147162 21847380

[51] van Groen T, and Michael Wyss J (1990). Connections of the retrosplenial granular a cortex in the rat. J. Comp. Neurol. 300, 593–606. 10.1002/cne.903000412. 2273095

[52] van Groen T, and Wyss JM (1992). Connections of the retrosplenial dysgranular cortex in the rat. J. Comp. Neurol. 315, 200–216. 10.1002/cne.903150207. 1545009

[53] Vann SD, Aggleton JP, and Maguire EA (2009). What does the retrosplenial cortex do? Nat. Rev. Neurosci. 10, 792–802. 10.1038/nrn2733. 19812579

[54] Li Y, Ren M, Liu B, Jiang T, Jia X, Zhang H, Gong H, and Wang X (2025). Dissection of the long-range circuit of the mouse intermediate retrosplenial cortex. Commun. Biol. 8, 56. 10.1038/s42003-025-07463-8. PMC11736107 39814996

[55] Miller AMP, Vedder LC, Law LM, and Smith DM (2014). Cues, context, and long-term memory: the role of the retrosplenial cortex in spatial cognition. Front. Hum. Neurosci. 8. 10.3389/fnhum.2014.00586. PMC4122222 25140141

[56] McGeorge AJ, and Faull RLM (1989). The organization of the projection from the cerebral cortex to the striatum in the rat. Neuroscience 29, 503–537. 10.1016/0306-4522(89)90128-0. 2472578

[57] Foster GA, Sizer AR, Rees H, and Roberts MHT (1989). Afferent projections to the rostral anterior pretectal nucleus of the rat: a possible role in the processing of noxious stimuli. Neuroscience 29, 685–694. 10.1016/0306-4522(89)90141-3. 2472580

[58] Tonegawa S, Liu X, Ramirez S, and Redondo R (2015). Memory Engram Cells Have Come of Age. Neuron 87, 918–931. 10.1016/j.neuron.2015.08.002. 26335640

[59] Josselyn SA, and Tonegawa S (2020). Memory engrams: Recalling the past and imagining the future. Science 367, eaaw4325. 10.1126/science.aaw4325. PMC7577560 31896692

[60] Kitamura T, Ogawa SK, Roy DS, Okuyama T, Morrissey MD, Smith LM, Redondo RL, and Tonegawa S (2017). Engrams and circuits crucial for systems consolidation of a memory. Science 356, 73–78. 10.1126/science.aam6808. PMC5493329 28386011

[61] Ryan TJ, and Frankland PW (2022). Forgetting as a form of adaptive engram cell plasticity. Nat. Rev. Neurosci. 23, 173–186. 10.1038/s41583-021-00548-3. 35027710

[62] Corcoran KA, Donnan MD, Tronson NC, Guzmán YF, Gao C, Jovasevic V, Guedea AL, and Radulovic J (2011). NMDA Receptors in Retrosplenial Cortex Are Necessary for Retrieval of Recent and Remote Context Fear Memory. J. Neurosci. 31, 11655–11659. 10.1523/jneurosci.2107-11.2011. PMC3159389 21832195

[63] Czajkowski R, Jayaprakash B, Wiltgen B, Rogerson T, Guzman-Karlsson MC, Barth AL, Trachtenberg JT, and Silva AJ (2014). Encoding and storage of spatial information in the retrosplenial cortex. Proc. Natl. Acad. Sci. 111, 8661–8666. 10.1073/pnas.1313222111. PMC4060653 24912150

[64] Zingg B, Peng B, Huang J, Tao HW, and Zhang LI (2020). Synaptic Specificity and Application of Anterograde Transsynaptic AAV for Probing Neural Circuitry. J. Neurosci. 40, 3250–3267. 10.1523/jneurosci.2158-19.2020. PMC7159884 32198185

[65] Yu AL, and DeNardo LA (2025). Long-Term Memory Engrams From Development to Adulthood. Hippocampus 35, e70032. 10.1002/hipo.70032. PMC12326896 40767385

[66] Katche C, Dorman G, Gonzalez C, Kramar CP, Slipczuk L, Rossato JI, Cammarota M, and Medina JH (2013). On the role of retrosplenial cortex in long-lasting memory storage. Hippocampus 23, 295–302. 10.1002/hipo.22092. 23355414

[67] Power SD, Stewart E, Zielke LG, Byrne EP, Douglas A, Ortega-de San Luis C, Lynch L, and Ryan TJ (2023). Immune activation state modulates infant engram expression across development. Sci. Adv. 9, eadg9921. 10.1126/sciadv.adg9921. PMC10631722 37939176

[68] Veldman MB, Park CS, Eyermann CM, Zhang JY, Zuniga-Sanchez E, Hirano AA, Daigle TL, Foster NN, Zhu M, Langfelder P, (2020). Brainwide Genetic Sparse Cell Labeling to Illuminate the Morphology of Neurons and Glia with Cre-Dependent MORF Mice. Neuron 108, 111–127.e6. 10.1016/j.neuron.2020.07.019. PMC7572760 32795398

[69] Sorra KE, and Harris KM (2000). Overview on the structure, composition, function, development, and plasticity of hippocampal dendritic spines. Hippocampus 10, 501–511. 10.1002/1098-1063(2000)10:5<501::aid-hipo1>3.0.co;2-t. 11075821

[70] Jacot-Descombes S, Keshav NU, Dickstein DL, Wicinski B, Janssen WGM, Hiester LL, Sarfo EK, Warda T, Fam MM, Harony-Nicolas H, (2020). Altered synaptic ultrastructure in the prefrontal cortex of Shank3-deficient rats. Mol. Autism 11, 89. 10.1186/s13229-020-00393-8. PMC7671669 33203459

[71] Bourne J, and Harris KM (2007). Do thin spines learn to be mushroom spines that remember? Curr. Opin. Neurobiol. 17, 381–386. 10.1016/j.conb.2007.04.009. 17498943

[72] Harris KM (1999). Structure, development, and plasticity of dendritic spines. Curr. Opin. Neurobiol. 9, 343–348. 10.1016/s0959-4388(99)80050-6. 10395574

[73] Bessières B, Prikas E, Goodwin-Groen S, Silenzi L, and Alberini CM (2026). Infant learning forms lasting memory schemas that influence adult behavior. Neuron S0896627326002096. 10.1016/j.neuron.2026.03.018. PMC13078708 41966691

[74] Yadav N, Toader A, and Rajasethupathy P (2024). Beyond hippocampus: Thalamic and prefrontal contributions to an evolving memory. Neuron 112, 1045–1059. 10.1016/j.neuron.2023.12.021. PMC12945457 38272026

[75] Keene CS, and Bucci DJ (2008). Neurotoxic lesions of retrosplenial cortex disrupt signaled and unsignaled contextual fear conditioning. Behav. Neurosci. 122, 1070–1077. 10.1037/a0012895. 18823164

[76] Frank AC, Huang S, Zhou M, Gdalyahu A, Kastellakis G, Silva TK, Lu E, Wen X, Poirazi P, Trachtenberg JT, and Silva AJ (2018). Hotspots of dendritic spine turnover facilitate clustered spine addition and learning and memory. Nat. Commun. 9, 422. 10.1038/s41467-017-02751-2. PMC5789055 29379017

[77] Yashiro K, and Philpot BD (2008). Regulation of NMDA receptor subunit expression and its implications for LTD, LTP, and metaplasticity. Neuropharmacology 55, 1081–1094. 10.1016/j.neuropharm.2008.07.046. PMC2590778 18755202

[78] Wu MW, Kourdougli N, and Portera-Cailliau C (2024). Network state transitions during cortical development. Nat. Rev. Neurosci. 25, 535–552. 10.1038/s41583-024-00824-y. PMC11825063 38783147

[79] Wang W, Schuette PJ, Nagai J, Tobias BC, Cuccovia V Reis, F.M., Ji, S., de Lima, M.A., La-Vu, M.Q., Maesta-Pereira, S., Chakerian, M., (2021). Coordination of escape and spatial navigation circuits orchestrates versatile flight from threats. Neuron 109, 1848–1860.e8. 10.1016/j.neuron.2021.03.033. PMC8178241 33861942

[80] Roy DS, Zhang Y, Aida T, Choi S, Chen Q, Hou Y, Lea NE, Skaggs KM, Quay JC, Liew M, (2021). Anterior thalamic dysfunction underlies cognitive deficits in a subset of neuropsychiatric disease models. Neuron 109, 2590–2603.e13. 10.1016/j.neuron.2021.06.005. PMC8376805 34197733

[81] Klune CB, Goodpaster CM, Gongwer MW, Gabriel CJ, An J, Chen R, Jones NS, Williams OH, Shari M, Ramirez M, (2025). Developmentally distinct architectures in top–down pathways controlling threat avoidance. Nat. Neurosci. 28, 823–835. 10.1038/s41593-025-01890-w. PMC11978489 39972221

[82] Baumgärtel K, Green A, Hornberger D, Lapira J, Rex C, Wheeler DG, and Peters M (2018). PDE4D regulates Spine Plasticity and Memory in the Retrosplenial Cortex. Sci. Rep. 8, 3895. 10.1038/s41598-018-22193-0. PMC5832851 29497131

[83] Greer PL, and Greenberg ME (2008). From Synapse to Nucleus: Calcium-Dependent Gene Transcription in the Control of Synapse Development and Function. Neuron 59, 846–860. 10.1016/j.neuron.2008.09.002. 18817726

[84] Warren DE, Rangel AJ, Christopher-Hayes NJ, Eastman JA, Frenzel MR, Stephen JM, Calhoun VD, Wang Y, and Wilson TW (2021). Resting-state functional connectivity of the human hippocampus in periadolescent children: Associations with age and memory performance. Hum. Brain Mapp. 42, 3620–3642. 10.1002/hbm.25458. PMC8249892 33978276

[85] Mathis A, Mamidanna P, Cury KM, Abe T, Murthy VN, Mathis MW, and Bethge M (2018). DeepLabCut: markerless pose estimation of user-defined body parts with deep learning. Nat. Neurosci. 21, 1281–1289. 10.1038/s41593-018-0209-y. 30127430

[86] Gabriel CJ, Zeidler Z, Jin B, Guo C, Goodpaster CM, Kashay AQ, Wu A, Delaney M, Cheung J, DiFazio LE, (2022). BehaviorDEPOT is a simple, flexible tool for automated behavioral detection based on markerless pose tracking. eLife 11, e74314. 10.7554/elife.74314. PMC9398447 35997072

[87] Chi J, Crane A, Wu Z, and Cohen P (2018). Adipo-clear: A tissue clearing method for three-dimensional imaging of adipose tissue. J. Vis. Exp. 2018, 58271. 10.3791/58271 PMC6126572 30102289

[88] Gongwer MW, Klune CB, Couto J, Jin B, Enos AS, Chen R, Friedmann D, and DeNardo LA (2023). Brain-Wide Projections and Differential Encoding of Prefrontal Neuronal Classes Underlying Learned and Innate Threat Avoidance. J. Neurosci. 43, 5810–5830. 10.1523/jneurosci.0697-23.2023. PMC10423051 37491314

[89] Friedmann D, Pun A, Adams EL, Lui JH, Kebschull JM, Grutzner SM, Castagnola C, Tessier-Lavigne M, and Luo L (2020). Mapping mesoscale axonal projections in the mouse brain using a 3D convolutional network. Proc. Natl. Acad. Sci. USA 117, 11068–11075. 10.1073/pnas.1918465117. PMC7245124 32358193

[90] Wang Q, Ding SL, Li Y, Royall J, Feng D, Lesnar P, Graddis N, Naeemi M, Facer B, Ho A, (2020). The Allen Mouse Brain Common Coordinate Framework: A 3D Reference Atlas. Cell 181, 936– 953.e20. 10.1016/j.cell.2020.04.007. PMC8152789 32386544

[91] Carey H, Pegios M, Martin L, Saleeba C, Turner AJ, Everett NA, Bjerke IE, Puchades MA, Bjaalie JG, and McMullan S (2023). DeepSlice: rapid fully automatic registration of mouse brain imaging to a volumetric atlas. Nat. Commun. 14, 5884. 10.1038/s41467-023-41645-4. PMC10514056 37735467

[92] Chiaruttini N, Burri O, Haub P, Guiet R, Sordet-Dessimoz J, and Seitz A (2022). An Open-Source Whole Slide Image Registration Workflow at Cellular Precision Using Fiji, QuPath and Elastix. Front. Comput. Sci. 3, 780026. 10.3389/fcomp.2021.780026.

[93] Schindelin J, Arganda-Carreras I, Frise E, Kaynig V, Longair M, Pietzsch T, Preibisch S, Rueden C, Saalfeld S, Schmid B, (2012). Fiji: an open-source platform for biological-image analysis. Nat. Methods 9, 676–682. 10.1038/nmeth.2019. PMC3855844 22743772

[94] Risher WC, Ustunkaya T, Singh Alvarado J, and Eroglu C (2014). Rapid Golgi Analysis Method for Efficient and Unbiased Classification of Dendritic Spines. PLoS One 9, e107591. 10.1371/journal.pone.0107591. PMC4160288 25208214

[95] Krishnan A, Williams LJ, McIntosh AR, and Abdi H (2011). Partial Least Squares (PLS) methods for neuroimaging: A tutorial and review. Neuroimage 56, 455–475. 10.1016/j.neuroimage.2010.07.034. 20656037

[96] Franceschini A, Mazzamuto G, Checcucci C, Chicchi L, Fanelli D, Costantini I, Passani MB, Silva BA, Pavone FS, and Silvestri L (2023). Brain-wide neuron quantification toolkit reveals strong sexual dimorphism in the evolution of fear memory. Cell Rep. 42, 112908. 10.1016/j.celrep.2023.112908. 37516963

