# Supplementary material for: Retrosplenial cortex maturation aligns with the transition from infant forgetting to persistent memory
Source: Cell Rep. Author manuscript; Available in PMC 2026 Oct 13. (PMC13661129; doi:10.1016/j.celrep.2026.117768)
Supplement: Supplementary file 1 [file NIHMS2212215-supplement-1.pdf]

Cell Reports, Volume *45*

Supplemental information

Retrosplenial cortex maturation aligns with the transition from infant forgetting to persistent memory

Benita Jin, Abigail L. Yu, Michael W. Gongwer, Brian P. Kearney, Alfonso Darmawan, Lilit Ohanian, Lucinda Holden-Wingate, Bryan Le, Yuka Nakayama, Sophia A. Rueda Mora, Anish Velaparthi, and Laura A. DeNardo

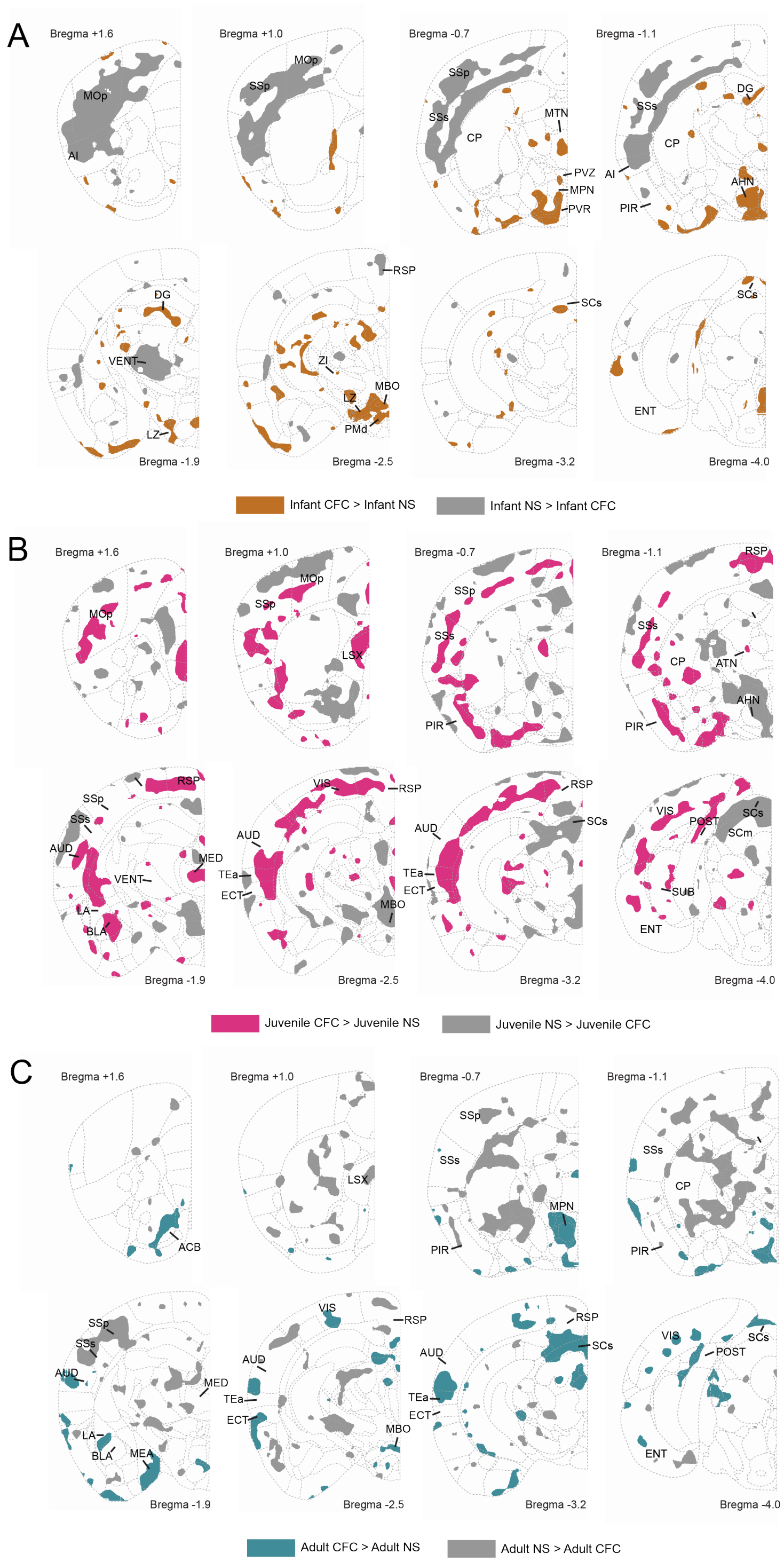

Figure S1. Voxel-based analysis comparing learning indices in infant CFC to age-matched non-shock controls, Related to Figure 1. Values from each voxel underwent an unpaired two-tailed student's t-test. Colored voxels, $p < 0.05$ for the comparison indicated in the legend.

(A) Infant (N = 5 CFC, N = 4 non-shock).

(B) Juvenile (N = 4 CFC, N = 7 non-shock).

(C) Adult (N = 4 CFC, N = 7 non-shock).

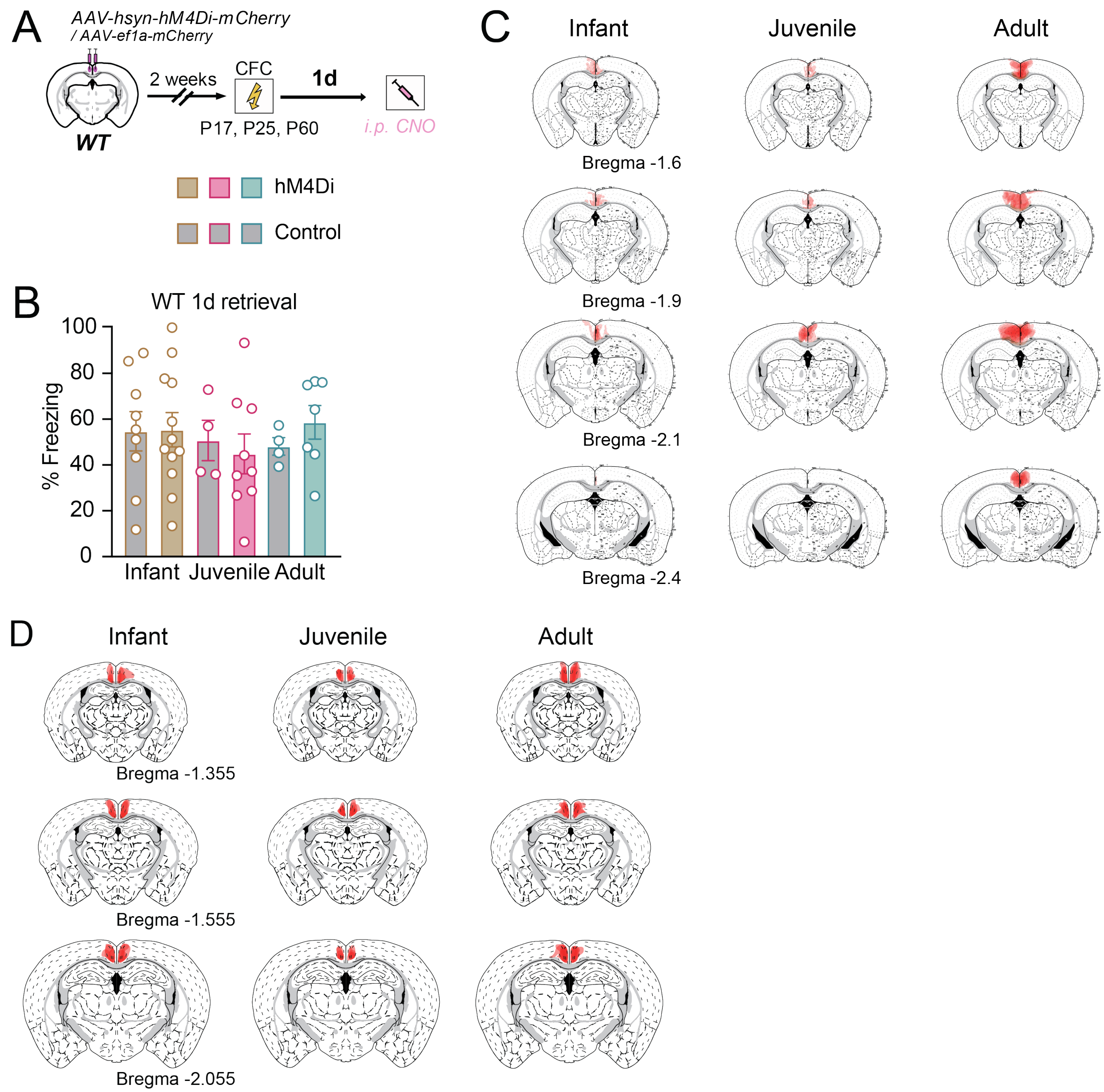

Figure S2. Inhibition of RSP at 1d retrieval in WT mice. Related to Figure 2.
(A) Experimental design.
(B) Freezing during 1d retrieval for hM4Di (color filled) vs controls (grey filled) in infant (brown), juveniles (pink), and adult (teal) WT mice. Data presented as mean ± SEM.
(C) Mapping viral targeting of AAV-hM4Di.
(D) Coronal sections showing RSP hM3Dq-mCherry expression in CFC-TRAPed mice

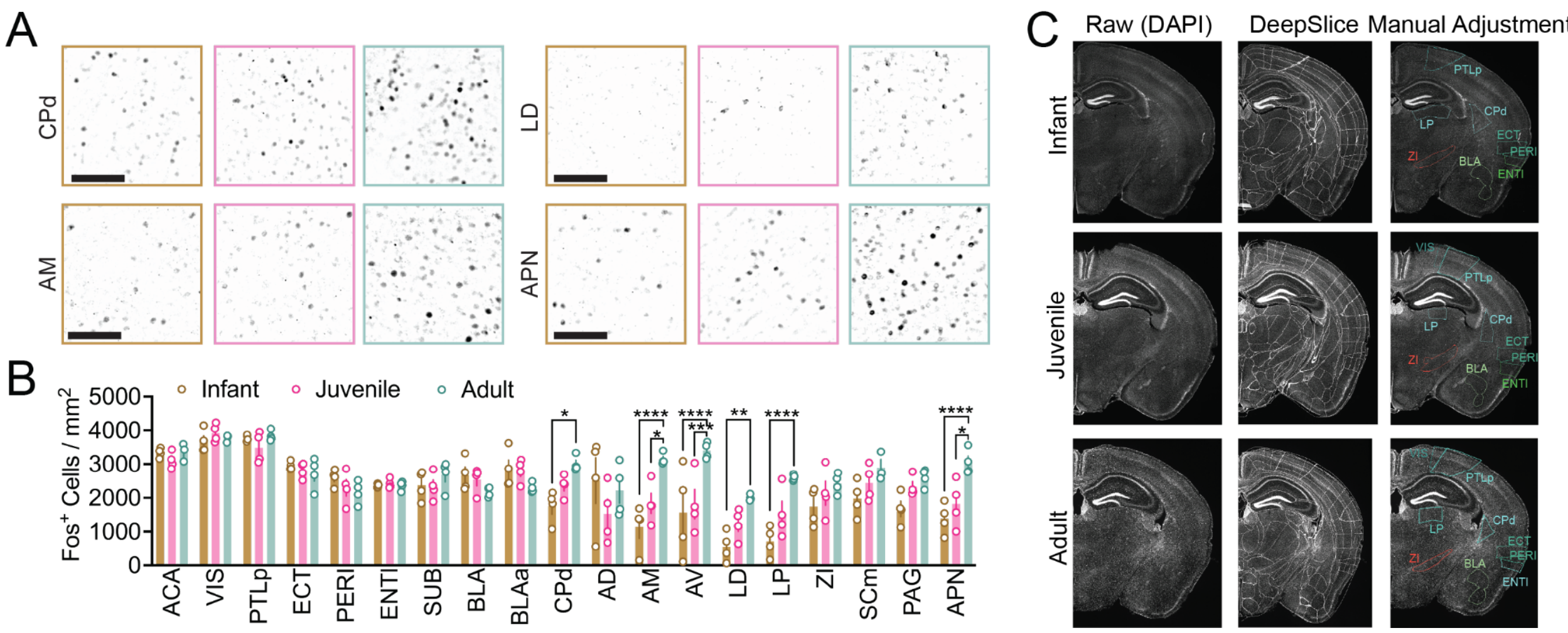

Figure S3. Fos+ expression in regions projecting to RSP after 7d retrieval. Related to Figure 2, Table 1.

(A) Representative images showing Fos immunoreactive cells in dorsal caudate putamen (CPd), anteromedial thalamus (AM), laterodorsal thalamus (LD), and anterior pretectal nucleus (APN). Scale bars, 100 µm.

(B) Quantification of Fos+ cells per ROI in 19 brain regions ($F_{region}(18,160)=30.03$, $p < 0.05$; $F_{age}(2,9)=3.69$, $p > 0.05$; $F_{int}(36,160)=5.78$, $p < 0.05$, Mixed Effects Model; Infant: $N = 4$; Juvenile: $N = 4$; Adult: $N = 3$-4).

(C) Representative atlas alignments for each age group.

Data presented as mean ± SEM. * $p < 0.05$, ** $p < 0.01$, *** $p < 0.001$, **** $p < 0.0001$. See also Figure S4 and S5. See Table 1 for abbreviations.

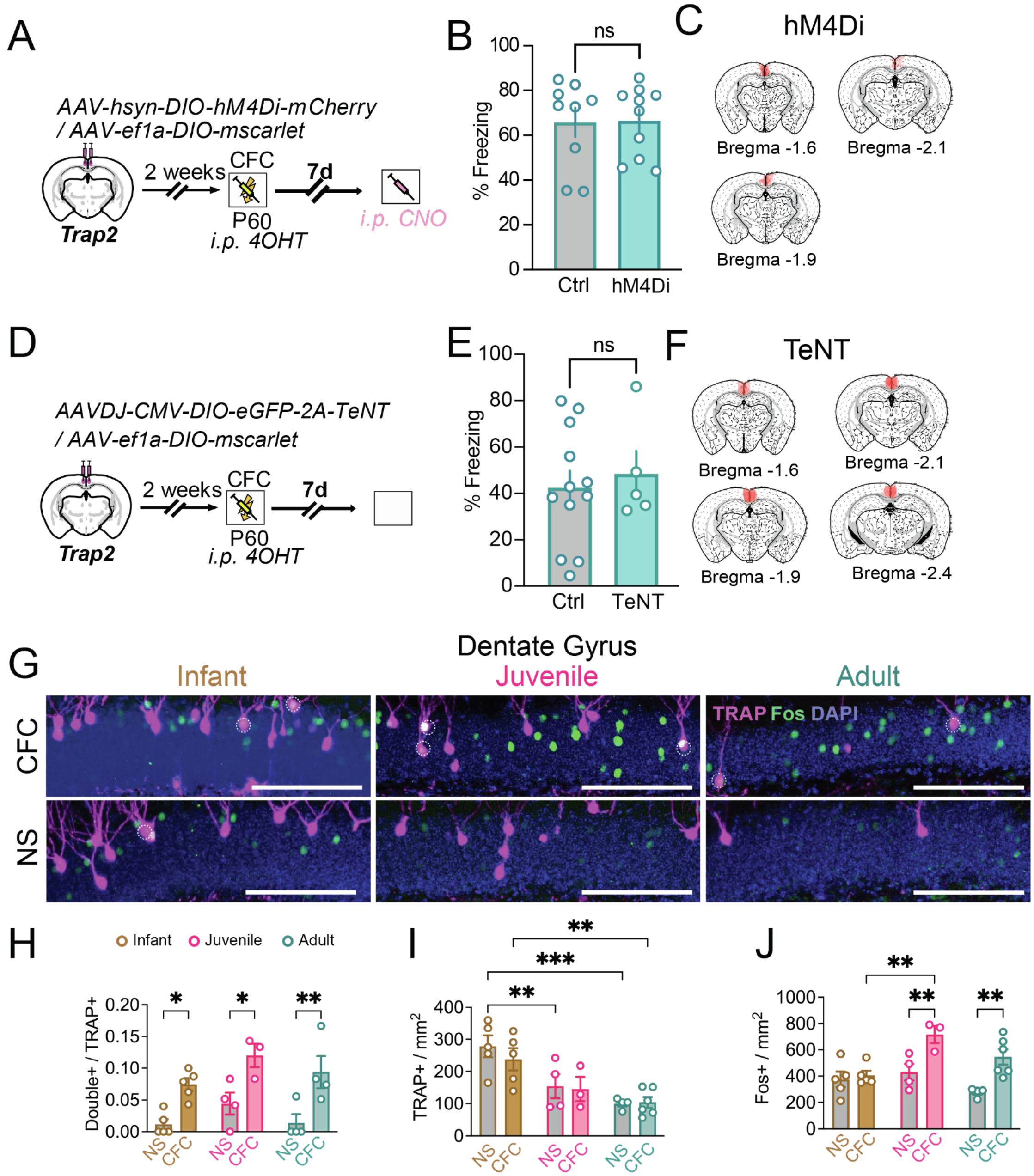

Figure S4. Inhibition of adult RSP. Related to Figure 3.

(A) Experimental design for chemogenetic inhibition of TRAP cells at 7d retrieval.

(B) Percent freezing during 7d retrieval for control (grey) and hM4Di (teal) adult animals. CNO was injected 30 minutes before the start of the retrieval session (p = 0.928, unpaired t test).

(C) Coronal sections showing location of RSP hM4Di-mCherry expression in adult-TRAPed mice.

(D) Experimental design for TeNT inhibition of synaptic output from TRAP cells.

(E) Freezing during the 7d retrieval session. (p = 0.6344, unpaired t test with Welch's correction)

(F) Coronal sections showing location of RSP TeNT-mCherry expression in adult TRAPed cells.

(G) Representative examples of TRAPed (magenta) and Fos+ cells (green) in dentate gyrus (DG) after CFC (top) or in NS control mice (bottom). Double-labeled cells are in white.

(H) Reactivated neurons in DG after CFC/NS and 7d retrieval ($F_{shock}(1,19)=32.56$, p = 0.0001, $F_{age}(2,19)=3.19$, p = 0.064; $F_{int}(2,19)=0.18$, p = 0.83; two-way ANOVA with Sidak post-hoc

correction; Infant: N = 5 CFC, N = 5 NS; Juvenile: N = 3 CFC, N = 4 NS; Adult: N = 4 CFC, N = 4 NS mice).

(I) Reactivated TRAPed DG cells after 7d retrieval ($F_{shock}(1,21)=0.37$, $p = 0.55$, $F_{age}(2,21)=15.89$, $p < 0.0001$; $F_{int}(2,21)=0.33$, $p = 0.73$; two-way ANOVA with Tukey post-hoc correction; Infant: N = 5 CFC, N = 5 NS; Juvenile: N = 3 CFC, N = 4 NS; Adult: N = 6 CFC, N = 4 NS mice).

(J) Fos+ DG cells after 7d retrieval ($F_{shock}(1,21)=19.14$, $p = 0.0003$, $F_{age}(2,21)=5.97$, $p = 0.009$; $F_{int}(2,21)=3.64$, $p = 0.04$; two-way ANOVA with Tukey post-hoc correction; Infant: N = 5 CFC, N = 5 NS; Juvenile: N = 7 CFC, N = 4 NS; Adult: N = 6 CFC, N = 4 NS mice).

Data presented as mean ± SEM. * $p<0.05$, ** $p<0.01$, *** $p<0.001$. Scale bar, 100 μm.

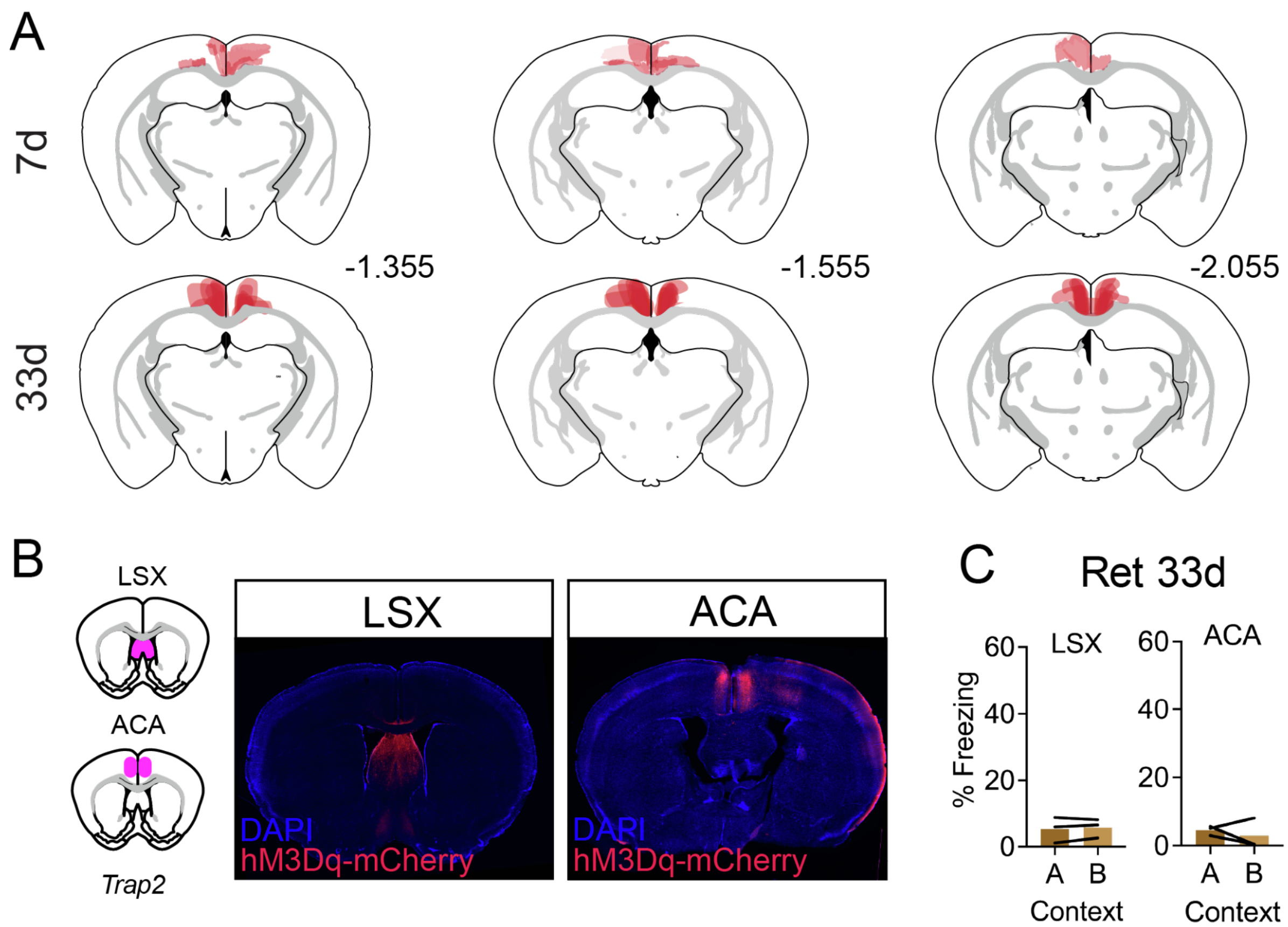

Figure S5. Inhibiting TRAPed cells in LSX and ACA weeks after CFC and mapping viral targeting in RSP, Related to Figure 5.
(A) Coronal sections showing location of RSP hM3D-mCherry expression in infant-TRAPed mice after 7d or 33d retrieval.
(B) Representative coronal section showing infant CFC-TRAPed cells expressing AAV-hM3Dq in LSX and ACA.
(C) Percent freezing levels in contexts A and B during the 33d retrieval test in LSX- and ACA-targeted mice (LSX: $p = 0.54$, N = 3, ACA: $p = 0.56$, N = 3, paired t-test). Data presented as mean with individual data points.
